# Integrating mandibular evidence to assess morphological variation of the *Australopithecus afarensis* maxilla

**DOI:** 10.1002/ar.70027

**Published:** 2025-07-31

**Authors:** Hester Hanegraef, Romain David, Fred Spoor

**Affiliations:** ^1^ Centre for Human Evolution Research Natural History Museum London UK; ^2^ Department of Human Origins Max Planck Institute for Evolutionary Anthropology Leipzig Germany

**Keywords:** dental arcade, predictions, sexual dimorphism, temporal trends, variation

## Abstract

Geometric morphometric analyses are used to explore variation of maxillary dental arcades of *Australopithecus afarensis*, expanding on the work of Hanegraef and Spoor, 2025 (Morphological variation of the *Australopithecus afarensis* maxilla. *Journal of Human Evolution*, *201*, 103651) by integrating evidence from a large sample of virtually reconstructed mandibles. Size and shape of maxillary dental arcades can be predicted accurately from mandibular landmarks based on strong covariation between occluding upper and lower dentitions, and a novel method was developed to correct for reduced shape variation in these predictions. As predictions are restricted to the alveolar process, morphological information about the rest of the maxilla is lost. The trade‐off between a smaller sample with comprehensive morphology and a larger sample with restricted morphology is discussed. Here, we analyzed 9 original and 17 predicted *A. afarensis* dental arcades in the comparative context of 448 extant hominine (modern human and African ape) maxillae. This study found that (1) degrees of size and shape variation are high in *A. afarensis*, potentially even higher than in *Gorilla* species when including the predictions in the fossil sample, (2) no allometry was detected, even when expanding the *A. afarensis* sample with predictions, (3) size and shape do not significantly change over time when analyzing original and predicted *A. afarensis* dental arcades together, and (4) sexual form and shape dimorphism, but not sexual size differences, are reduced when including *A. afarensis* predictions in the fossil sample. Our results quantifying the range and pattern of variation of the *A. afarensis* maxilla provide a comparative context when assessing whether or not other Plio‐Pleistocene hominin specimens are conspecific.

## 1 INTRODUCTION

A detailed morphological study of the maxilla of *Australopithecus afarensis* showed that this species has high degrees of size and shape variation compared with extant hominine (modern human and African ape) species (Hanegraef & Spoor, 2025). Although *A. afarensis* is among the best represented early hominin taxa in the fossil record, the study was nevertheless restricted to nine sufficiently preserved maxillae from Hadar. While this sample provides valuable insights, it is still limited by its sample size, especially as it represents a time depth of approximately 500,000 years. A likely taphonomic factor resulting in underrepresentation of maxillae in the fossil record is that this part of the skull is relatively fragile, with thin bone forming the walls of the maxillary sinuses, the nasal cavity, and the orbital socket. In contrast, the mandible is the most common element other than teeth in the *A. afarensis* hypodigm (Kimbel & Delezene, 2009), reflecting its robust architecture, particularly of the corpus.

Studying maxillae, it is possible to take advantage of the relative abundance of mandibles by using the latter's dental arcade morphology to infer the associated maxillary dental arcade for each individual on the basis of the strong covariation between the occluding upper and lower dentitions (Spoor et al., 2015; Stelzer et al., 2017, 2018). Using mandibular evidence thus substantially increases sample sizes and adds to the overall understanding of maxillary variation, but predicted data will inevitably be less accurate than when collected from maxillae directly. Moreover, the extra information is restricted to the alveolar process of the maxilla, yet it nevertheless expresses a plethora of biologically relevant characteristics, including the dental arcade size and shape, its anterior projection, and proportions of alveolar segments representing incisors, canines, premolars, and molars.

Predictions of fossil dental arcades are obtained using regression models derived from extant hominine species. The reference sample therefore does not include individuals of the taxon for which the prediction is made, a special case of the exclusion model as defined by Stelzer et al. (2018). Nonetheless, assuming that fossil hominins exhibit a pattern of covariation between their upper and lower dental arcades most similar among extant taxa to modern humans and African apes, a reference sample including these species is appropriate. This holds even if overall dental arcade shape differs between the fossil and extant samples, as the key assumption here concerns the conservation of the covariation relationship.

The specific aims of this research are (1) to explore the pattern and magnitude of covariation between the maxillary and mandibular dental arcade of extant hominine species, (2) to quantify and evaluate the accuracy of predicting complementary arcades in this context, (3) to use these methods to predict maxillary dental arcades from *A. afarensis* mandibles, (4) to explore the degree and nature of size and shape variation in the maxillary dental arcades of *A. afarensis*, including actual and predicted arcades, in the comparative context of variation in extant hominine species, (5) to assess the role of intraspecific static allometry in shape differences, (6) to examine temporal trends in the *A. afarensis* maxillary dental arcade morphology, (7) to investigate the degree and nature of sexual dimorphism, and (8) to determine the impact of including late juveniles in the fossil sample.

## 2 MATERIALS AND METHODS

### 2.1 Sample

The fossil sample consists of all sufficiently preserved *A. afarensis* jaws from Hadar and Laetoli (Table 1), including the mandibles of 19 adults and one late juvenile, as well as the maxillae of eight adults and one late juvenile described in Hanegraef and Spoor (2025). Three pairs of maxillae and mandibles are from the same fossil individuals (A.L. 417‐1, A.L. 444‐2, and A.L. 822‐1). L.H. 4 is completely described in White (1977), A.L. 288‐1i in Johanson, Lovejoy, et al. (1982), A.L. 199‐1 and A.L. 200‐1a in Kimbel et al. (1982), A.L. 128‐23, A.L. 198‐1, A.L. 266‐1, A.L. 277‐1, A.L. 333w‐60, and A.L. 400‐1a in White and Johanson (1982), A.L. 315‐22, A.L. 330‐5, A.L. 437‐1, A.L. 437‐2, A.L. 438‐1g, and A.L. 620‐1 in Robinson (2003), and A.L. 444‐2 in Kimbel et al. (2004). An initial description for A.L. 417‐1 is provided by Kimbel et al. (1994), with the mandible more comprehensively described in Robinson (2003). An initial description of A.L. 822‐1 can be found in Kimbel and Rak (2010). For A.L. 427‐1a, A.L. 442‐1, and A.L. 486‐1, no formal descriptions have yet been published, but specific features and illustrations of these specimens are presented in a comparative context in Kimbel et al. (2004) and Kimbel and Delezene (2009). A.L. 922‐1, A.L. 1045‐1, A.L. 1496‐1, and A.L. 1901‐1 are currently undescribed.

**TABLE 1:** Fossil sample size and composition.

No.	Specimen	Maxilla	Mandible	Age	Sex		Location	Stratigraphic unit	Missing landmarks^p^
					Literature	This study			
1	A.L. 199‐1	●	○	A	F^d^	F	Hadar	SH‐1^d^	**1–9**, **13**
2	A.L. 200‐1a	●	○	A	M^b^, ^e^/F^f^	M	Hadar	SH‐1^d^	*22–24*
3	A.L. 417‐1d/ab	●	●	A	F^f^, ^g^	F	Hadar	SH‐3^f^	Maxilla: **1–16**
4	A.L. 427‐1a	●	○	A	M^f^	M	Hadar	DD‐3^f^	*13–16*, *28*
5	A.L. 442‐1	●	○	A	F^f^	F	Hadar	DD‐2^f^	**1–9**, **11–13**, **15–16**, **19**, **23**
6	A.L. 444‐2a/b	●	●	A	M^f^, ^g^, ^h^	M	Hadar	KH‐2^f^	—
7	A.L. 486‐1	●	○	LJ^a^, ^b^	M^b^	M	Hadar	DD‐3^f^	**19**, **23**
8	A.L. 822‐1	●	●	A	F^i^	F	Hadar	KH‐1^f^	—
9	A.L. 922‐1	●	○	A	M^b^	M	Hadar	KH‐2^f^	—
10	A.L. 128‐23	○	●	LJ^c^	F^g^, ^j^, ^k^	F	Hadar	SH‐1^d^	**1–9**, **13**
11	A.L. 198‐1	○	●	A	F^f^, ^k^	F	Hadar	SH‐1^d^	1–3, 5–7, 9, 13
12	A.L. 266‐1	○	●	A	F^f^, ^k^	F	Hadar	SH‐3^d^	**1–11**, **13–15**, **17–19**, **21–23**, *49–52*
13	A.L. 277‐1	○	●	A	M^f^, ^g^, ^j^, ^k^	M	Hadar	SH‐2^d^	**1–16**
14	A.L. 288‐1i	○	●	A	F^f^, ^g^, ^k^	F	Hadar	KH‐1^d^	**10–11**, *13*, **14–15**, *16*, **17**, **19**, **21**, **23**
15	A.L. 315‐22	○	●	A	F^f^, ^g^	F	Hadar	DD‐3^f^	1–17, 19–21, 23–24, 52, 56
16	A.L. 330‐5	○	●	A	F^f^	F	Hadar	SH‐4^f^	**1–3**, **5–7**, **9**, **11**, **13**, **15**, **27**, **31**
17	A.L. 333w‐60	○	●	A	M^e^, ^f^, ^j^, ^k^	M	Hadar	DD‐2^d^	—
18	A.L. 400‐1a	○	●	A	F?^f^, ^k^	F	Hadar	SH‐2^f^	*8*
19	A.L. 437‐1	○	●	A	M^f^, ^h^	M	Hadar	KH‐2^f^	—
20	A.L. 437‐2	○	●	A	M^f^, ^h^	M	Hadar	KH‐2^f^	—
21	A.L. 438‐1g	○	●	A	M^f^, ^g^, ^h^	M	Hadar	KH‐2^f^	1–9, 13, 41, 45
22	A.L. 620‐1	○	●	A	M^f^	M	Hadar	DD‐2^f^	**1**, **3** , **5**, **7** , **10**, **14**, **17**, **19** , **21**, **23**
23	A.L. 1045‐1	○	●	A	—	F	Hadar	SH‐2^m^	1–3, 5–7
24	A.L. 1496‐1	○	●	A	M^l^	M	Hadar	SH‐1^m^	**1–2**, **3** , **4–6**, **7** , **8–10**, **11** , **12–14**, **15** , **16–17**, **19** , **21**, *22*, **23** , *24*
25	A.L. 1901‐1	○	●	A	—	M	Hadar	DD‐3^n^	*5–7*, *13*, *15*, *27–28*, *49–52*
26	L.H. 4	○	●	A	M^f^, ^k^	M	Laetoli	ULB^o^	**1–16**, *17*, *19*

Abbreviations: A, adult; DD, Denen Dora; F, female; KH, Kada Hadar; LJ, late juvenile; M, male; SH, Sidi Hakoma; ULB, Upper Laetolil Beds; ●, present; ○, absent.

^a^ Dean et al. (2020).

^b^ Hanegraef and Spoor (2025).

^c^ White and Johanson (1982).

^d^ Johanson, Taieb, and Coppens (1982).

^e^ Kimbel et al. (1984).

^f^ Kimbel et al. (2004).

^g^ Kimbel et al. (1994).

^h^ Lockwood et al. (2000).

^i^ Kimbel et al. (2003) and Kimbel and Rak (2010).

^j^ Kimbel et al. (1985).

^k^ Leonard and Hegmon (1987).

^l^ Delezene and Kimbel (2011).

^m^ W. Kimbel (personal communication).

^n^ Conaway and Kimbel (2012).

^o^ Leakey et al. (1976).

^p^ See Table S3 for landmark numbers. Underlined landmarks are estimated using preserved morphology, italicized landmarks using bilateral symmetry, bold landmarks using thin‐plate spline interpolation, and bold‐underlined landmarks are manually adjusted after being estimated using thin‐plate spline interpolation.

With regards to the two late juveniles included in the fossil sample (Table 1), A.L. 128‐23 is dentally younger than A.L. 486‐1, with the M_3_ crypt exposed in the former (White & Johanson, 1982), while in the latter the M^3^ has erupted but is not yet in occlusion and has about half of its root length formed (Dean et al., 2020; Hanegraef & Spoor, 2025).

The L.H. 4 mandible was recovered from the Upper Laetolil Beds (Leakey et al., 1976), dated to ca. 3.85–3.63 Ma (Deino, 2011). The specimens from Hadar represent eight temporal groups based on their placement in the stratigraphic submembers of the Hadar Formation (Table 1) (Conaway & Kimbel, 2012; Johanson, Taieb, & Coppens, 1982; Kimbel et al., 2004; W. Kimbel, personal communication), dated between ca. 3.42 and 2.96 Ma (Campisano, 2007). Submember age ranges were taken from Campisano (2007) and specimens were plotted at the midpoint of those age brackets, with the exception of A.L. 288‐1i and A.L. 822‐1 from the KH‐1 submember, which are closer in age to 3.20 and 3.12 Ma, respectively (C. Campisano, personal communication).

Sex attributions of 24 *A. afarensis* jaws were obtained from published literature and are based on overall size, mandibular corpus height, and dental morphology and metrics, specifically of the canines, lower third premolars, and lower second molars (Table 1) (Delezene & Kimbel, 2011; Hanegraef & Spoor, 2025; Johanson, Taieb, & Coppens, 1982; Kimbel & Rak, 2010; Kimbel et al., 1984, 1985, 1994, 2003, 2004; Leonard & Hegmon, 1987; Lockwood et al., 2000). Additionally, evidence from associated cranial and postcranial elements has been used to determine the sex of A.L. 288‐1, A.L. 438‐1, A.L. 417‐1, A.L. 444‐2, and A.L. 822‐1 (Johanson & Taieb, 1976; Kimbel & Rak, 2010; Kimbel et al., 1994, 2003, 2004; Tobias, 1980). In the present study, sex was examined based on dental arcade size and the absolute width of the canine, third premolar, and second molar alveoli.

Currently known *A. afarensis* jaws from other localities were not included in this study due to incompleteness or unavailability. The DIK‐2‐1 mandible from Dikika (Alemseged et al., 2005) is preserved only up to the M_1_. Maxillae and mandibles from Maka (White et al., 2000) and Woranso‐Mille (Haile‐Selassie et al., 2016; Melillo et al., 2021) were not available at this time for our analysis.

The extant sample comprises 448 adult *Homo sapiens*, *Pan troglodytes*, *Pan paniscus*, *Gorilla gorilla*, and *Gorilla beringei* specimens as described in Hanegraef and Spoor (2025) and includes 369 matching pairs of maxillae and mandibles (Table S1, Supporting Information). A summary of the extant sample is provided in Table 2.

**TABLE 2:** Extant sample size and composition.

	Maxilla				Mandible			
	Total	M	F	U	Total	M	F	U
*H. sapiens*	100	57	33	10	74	40	25	9
*P. troglodytes*	100	50	50	—	91	46	45	—
*P. t. troglodytes*	30	14	16	—	25	11	14	—
*P. t. schweinfurthii*	35	19	16	—	33	18	15	—
*P. t. verus*	35	17	18	—	33	17	16	—
*P. paniscus*	47	21	26	—	44	18	26	—
*G. gorilla*	100	55	45	—	70	40	30	—
*G. g. gorilla*	78	47	31	—	68	40	28	—
*G. g. diehli*	22	8	14	—	2	0	2	—
*G. beringei*	101	56	45	—	90	52	38	—
*G. b. beringei*	25	14	11	—	20	13	7	—
*G. b. graueri*	76	42	34	—	70	39	31	—

Abbreviations: F, female; M, male; U, unknown sex.

### 2.2 CT scans and virtual reconstructions

Analyses are based on computed tomography (CT) scans. For the fossils, μCT scans were obtained at the National Museum of Ethiopia in Addis Ababa and the National Museum of Tanzania in Dar es Salaam, using the portable Skyscan 1173 μCT scanner of the Department of Human Evolution at the Max Planck Institute for Evolutionary Anthropology in Leipzig (isotropic voxel size: 0.03–0.07 mm). For the extant specimens, μCT scans (isotropic voxel size: 0.05–0.25 mm) and medical CT scans (pixel size: 0.15–0.71 mm, slice interval: 0.2–1.0 mm, slice thickness: 0.4–1.5 mm) were collected as described in Hanegraef and Spoor (2025).

Three‐dimensional (3D) digital surface visualizations were extracted from the CT scans and fossil specimens were virtually reconstructed using Avizo 7.1 (FEI Visualization Sciences Group, Berlin) as detailed in Hanegraef and Spoor (2025). Descriptions of the preservation and reconstruction procedure for the *A. afarensis* maxillae are presented in Hanegraef and Spoor (2025) and those for the *A. afarensis* mandibles are provided in Notes S1–S20 and illustrated in Figures S1–S24. When multiple reconstructions were created or reconstructions were less reliable, the impact was assessed (Note S21, Figure S25, and Table S2). The 20 reconstructed *A. afarensis* mandibles are shown in Figure 1.

**FIGURE 1:**
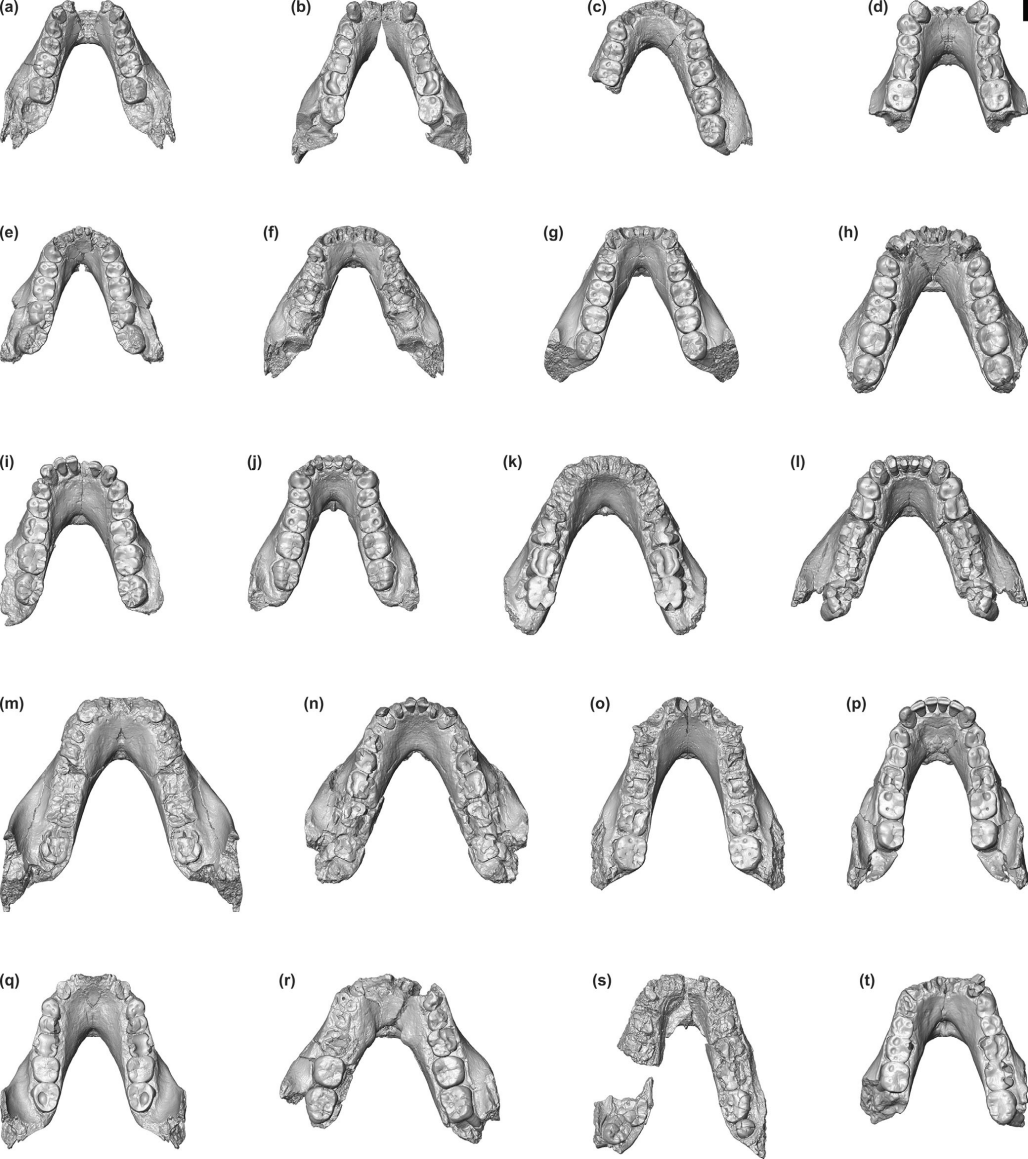
Occlusal view of the 20 reconstructed *A. afarensis* mandibles: A.L. 128‐23 (a), A.L. 198‐1 (b), A.L. 266‐1 (c), A.L. 277‐1 (d), A.L. 288‐1i (e), A.L. 315‐22 (f), A.L. 330‐5 (g), A.L. 333w‐60 (h), A.L. 400‐1a (i), A.L. 417‐1ab (j), A.L. 437‐1 (k), A.L. 437‐2 (l), A.L. 438‐1g (m), A.L. 444‐2b (n), A.L. 620‐1 (o), A.L. 822‐1 (p), A.L. 1045‐1 (q), A.L. 1496‐1 (r), A.L. 1901‐1 (s), and L.H. 4 (t). Scale bar 1 cm.

### 2.3 Landmark data

**FIGURE 2:**
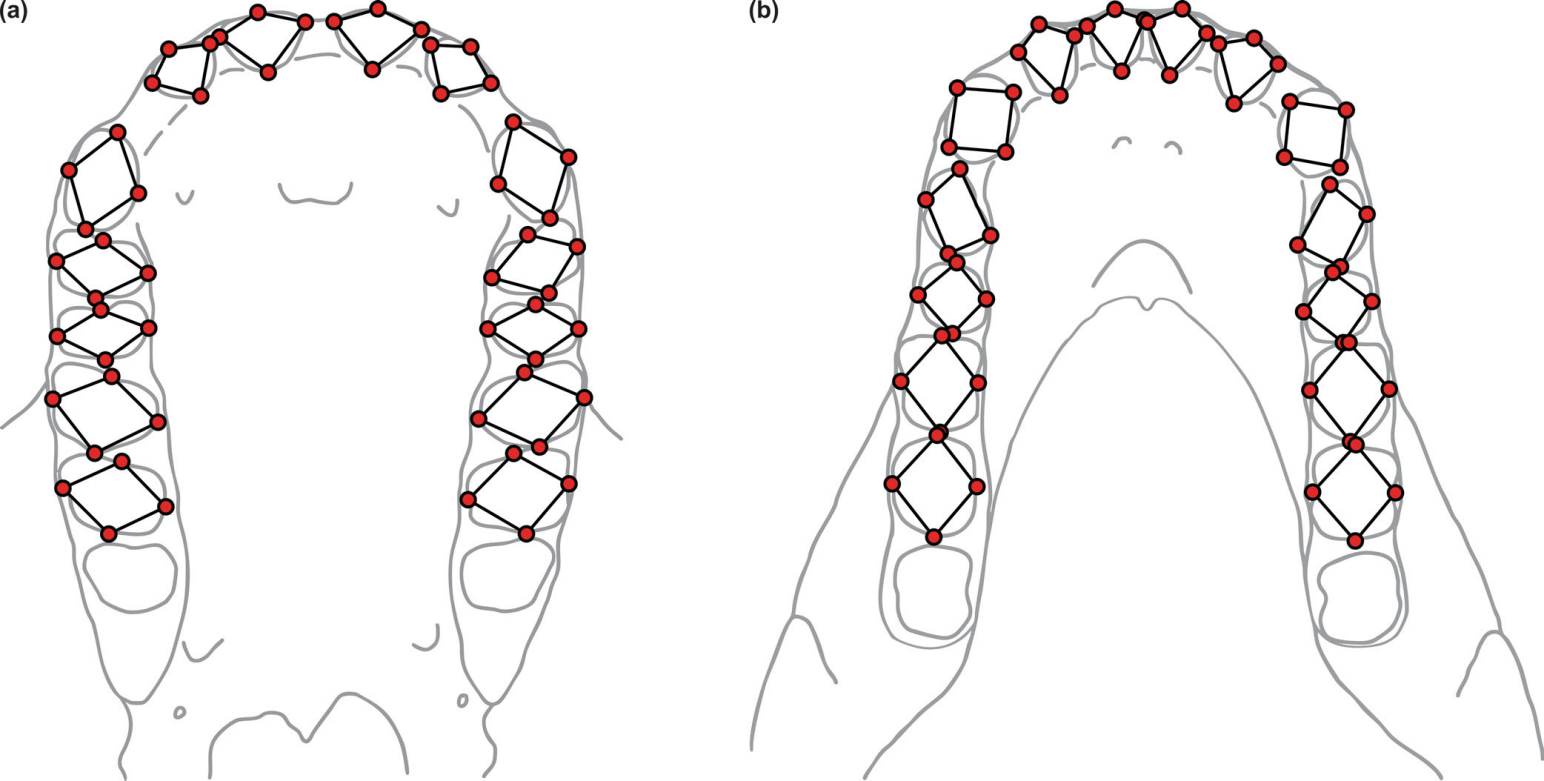
Landmarks (red circles) and wireframes (black lines) used to capture the shape of the maxillary (a) and mandibular (b) dental arcade, shown for a chimpanzee in occlusal view. Line drawings are based on USNM 176229.

The shape of each maxillary and mandibular dental arcade was captured by 56 3D landmarks (Figure 2 and Table S3) using Avizo 7.1. Landmarks were placed on the alveolar margins from the first incisor to the second molar on the left and right side. In contrast with the research of Stelzer et al. (2017, 2018, 2019), landmarks were not placed on the cervix of the postcanine dentition because crowns are often missing or badly preserved in fossil specimens. Landmarks were also not recorded for the third molars to allow the inclusion of late juveniles in the fossil sample. It was shown by Spoor et al. (2015) that comparative analyses of dental arcade shape are not meaningfully different with or without third molars.

Missing landmarks were estimated using morphological clues, bilateral symmetry, or thin‐plate spline interpolation separately for maxillae and mandibles (Gunz et al., 2009). In the latter case, specimens from the same species with a complete set of landmarks were used to obtain the reference specimen, as this should in principle produce the most accurate landmark predictions (Gunz et al., 2009; Senck et al., 2015; Zollikofer & de Ponce León, 2005). Landmarks that could not be measured on the surface scans of the *A. afarensis* specimens are listed in Table 1. The accuracy of landmark estimations has been determined in Hanegraef and Spoor (2025) for the *A. afarensis* maxillae and is discussed in Note S22 and illustrated in Figures S26–S28 for the *A. afarensis* mandibles.

All landmark data were symmetrized using reflected relabeling (Bookstein, 1991; Gunz et al., 2009; Mardia et al., 2000) and shape variables were obtained by applying a generalized Procrustes analysis separately to the maxillary and mandibular coordinates (Gower, 1975; Rohlf & Slice, 1990). Form variables were also acquired by adding the natural logarithms of centroid sizes as a separate variable to the shape variables (Dryden & Mardia, 1998; Kendall, 1989). Procrustes coordinates and natural logarithms of centroid sizes are available separately for the maxillae (Data S1) and the mandibles (Data S2).

### 2.4 Analyses

To investigate the pattern and magnitude of covariation between the maxillary and mandibular dental arcade (aim 1), size was assessed based on the centroid sizes of all extant hominine specimens for which upper and lower jaws are present. Statistical significance of the covariation in size was determined using the Pearson's product–moment correlation test. To assess taxonomic shape differences, principal component analyses were performed separately for the maxillary and mandibular shape variables. The pattern of shape covariation was then examined using a two‐block partial least squares analysis (Bookstein et al., 2003; Rohlf & Corti, 2000; Wold, 1966) and the magnitude of shape covariation was quantified for each extant hominine species by the correlation coefficient between the singular warp scores of each individual partial least square (Bookstein et al., 2003). These methods are similar to those applied in Stelzer et al. (2017), although sister species are sometimes pooled together in their analyses, and thus both intraspecific and intrageneric covariation are assessed. In this study, covariation is consistently examined at the species level.

Maxillary dental arcade sizes and shapes of extant hominine species were predicted from their complementary mandibles (aim 2), pooling all extant specimens for which both jaws are present and using leave‐one‐out cross validation so as not to bias the predictions (Stelzer et al., 2018). Centroid sizes of the maxillary dental arcades were predicted from the mandibular centroid sizes using a linear regression. Original and predicted natural logarithms of centroid sizes were plotted together for each extant species to visualize the differences. To statistically test the impact of predictions on intraspecific size differences and degrees of variation, the means and standard deviations of the natural logarithms of centroid sizes were compared between the original and predicted samples for each extant hominine species using pairwise *t* tests and pairwise *F* tests, respectively.

Maxillary dental arcade shapes were predicted from the mandibular landmark data of the extant hominine species. Following Spoor et al. (2015) and Stelzer et al. (2018, 2019), a multiple multivariate regression was used to predict the maxillary Procrustes coordinates from a subset of mandibular principal component (PC) scores. Here, the subspace of the first 10 PCs was used, which explains more than 95% of the total shape variance (Table S4). Predicted maxillae were projected into the shape space of the original maxillae to visualize the differences. To quantitatively assess these shape differences between the original and predicted data, Procrustes distances between the original and predicted dental arcade shapes were computed and then compared with the Procrustes shape distances between all possible specimen pairs for each extant hominine species using every available maxillary dental arcade. To visualize prediction error, the landmark configurations of the original arcades and their corresponding predicted arcades were plotted as wireframes, showing for each extant hominine species those individuals with the smallest and largest Procrustes shape distance between the original and predicted maxillary dental arcade. These methods investigating shape differences between original and predicted dental arcades are based on Spoor et al. (2015) and Stelzer et al. (2018), although neither study examined the effect of predictions on shape variation. Here, the degree of shape variation was assessed for the original and predicted sample through Procrustes distances between all possible specimen pairs for each extant species, and statistical significance was determined using pairwise *t* tests.

Maxillary dental arcades were then predicted from the *A. afarensis* mandibles (aim 3). Maxillary dental arcade sizes were predicted from the *A. afarensis* mandibular centroid sizes using the aforementioned linear regression, pooling all extant specimens with both jaws present. Original and predicted natural logarithms of centroid sizes were compared for the three *A. afarensis* specimens with associated maxillae and mandibles (A.L. 417‐1, A.L. 444‐2, and A.L. 822‐1).

Procrustes coordinates of maxillary dental arcades were predicted from the *A. afarensis* mandibular landmarks using the aforementioned multiple multivariate regression, pooling all extant specimens for which both jaws are present. Because predicted dental arcades tend to show reduced variation compared to the original data, we developed a new method to account for this loss of information in the fitted values. First, predicted maxillary dental arcades of extant hominine specimens were projected into the shape space of the original arcades. Residual errors were calculated by subtracting the projected PC scores of each predicted arcade from those of the corresponding original arcade. From these residuals, we created a multivariate normal distribution and extracted 1000 random samples. The predicted arcades of *A. afarensis* were then projected into the shape space of the original arcades of extant hominine specimens, and the randomly sampled residual errors were added to their projected PC scores. Procrustes coordinates were then predicted from these corrected PC scores, yielding 1000 predicted maxillary dental arcades for each *A. afarensis* specimen. Lastly, we computed the mean of these corrected predictions for each fossil specimen. The R code for the extant and fossil predictions, as well as this new protocol to correct predicted *A. afarensis* shapes is provided in Note S23.

The accuracy of corrected fossil predictions was assessed by comparing the Procrustes distance between the original shape and mean corrected prediction for the three *A. afarensis* specimens with associated maxillae and mandibles (A.L. 417‐1, A.L. 444‐2, and A.L. 822‐1), in the context of the Procrustes shape distances between all possible specimen pairs for each extant hominine species using every available maxillary dental arcade. Prediction uncertainty was then determined by calculating the Procrustes distances between the original shape and each of the 1000 corrected predictions for these three *A. afarensis* specimens. Prediction error was also visualized for the three specimens by comparing the landmark configuration of their original dental arcade shape with that of the mean corrected prediction.

The mean corrected predictions obtained from the *A. afarensis* mandibles were projected into the shape space of every original maxillary dental arcade to visualize prediction accuracy. Additionally, for each predicted fossil specimen, the Procrustes distances between the mean corrected shape and the 1000 corrected shapes were calculated, and a probability density was computed from these values. The Procrustes shape distance corresponding to the 69% quantile (1sd) of this distribution was calculated, and those corrected shapes whose Procrustes distance to the mean corrected shape was higher than the 69% quantile value were excluded from the sample. The remaining corrected predictions of each specimen were combined and projected into shape space to visualize the possible extent of the *A. afarensis* distribution. The same analyses were then performed to obtain the corrected predictions that fall within the 95% quantile (2sd).

The analyses to explore degrees of size and shape variation (aim 4), static allometry (aim 5), temporal trends (aim 6), and sexual dimorphism (aim 7) correspond to those outlined in Hanegraef and Spoor (2025). For *A. afarensis*, analyses were performed separately for the nine original maxillary dental arcades and for these nine plus the dental arcades predicted from the 17 mandibles without associated maxillae. In analyses that assess the shape of the *A. afarensis* dental arcade, one corrected shape of each predicted specimen was randomly sampled and analyzed together with the nine original maxillary dental arcade shapes of *A. afarensis* to account for prediction uncertainty. This process was repeated 1000 times and random sampling was done with replacement to obtain probabilities of statistical significance. Additionally, to examine the combined effects of size and sex on dental arcade shape, we tested four multivariate regression models separately for each extant hominine species: one including only size (shape ~ ln centroid size), a second including only sex (shape ~ sex), a third including both factors (shape ~ ln centroid size + sex), and a fourth including size, sex, and their interaction (shape ~ ln centroid size * sex). The fit of each model was then assessed based on the Akaike information criterion (AIC) values and their relative likelihood. Moreover, statistical significance of the temporal size trend in *A. afarensis* was assessed using the Pearson's product–moment correlation test.

Lastly, the impact of including late juveniles in the fossil sample (aim 8) was assessed by comparing the size and shape of A.L. 128‐23 and A.L. 486‐1 with those of the adult *A. afarensis* mandibles and maxillae, respectively.

All statistical analyses and visualizations were performed in R 4.5.0 (R Core Team, 2025), with the specific packages and functions provided in Table S5. All *p*‐values were adjusted for multiplicity, controlling the false discovery rate (Benjamini & Hochberg, 1995). Adjustments were applied separately for each variable, and the significance level was set at *p* < 0.05. All references to significant results concern statistically significant differences.

## 3 RESULTS

### 3.1 Pattern and magnitude of covariation (aim 1)

The covariation between maxillary and mandibular centroid sizes of extant hominine species is high and significant (*r* = 0.986, *t* = 114.859, *p* < 0.001). All specimens scatter along a diagonal when centroid sizes of corresponding upper and lower dental arcades are plotted against each other, although mandibles are proportionally smaller in larger specimens (Figure S29).

**FIGURE 3:**
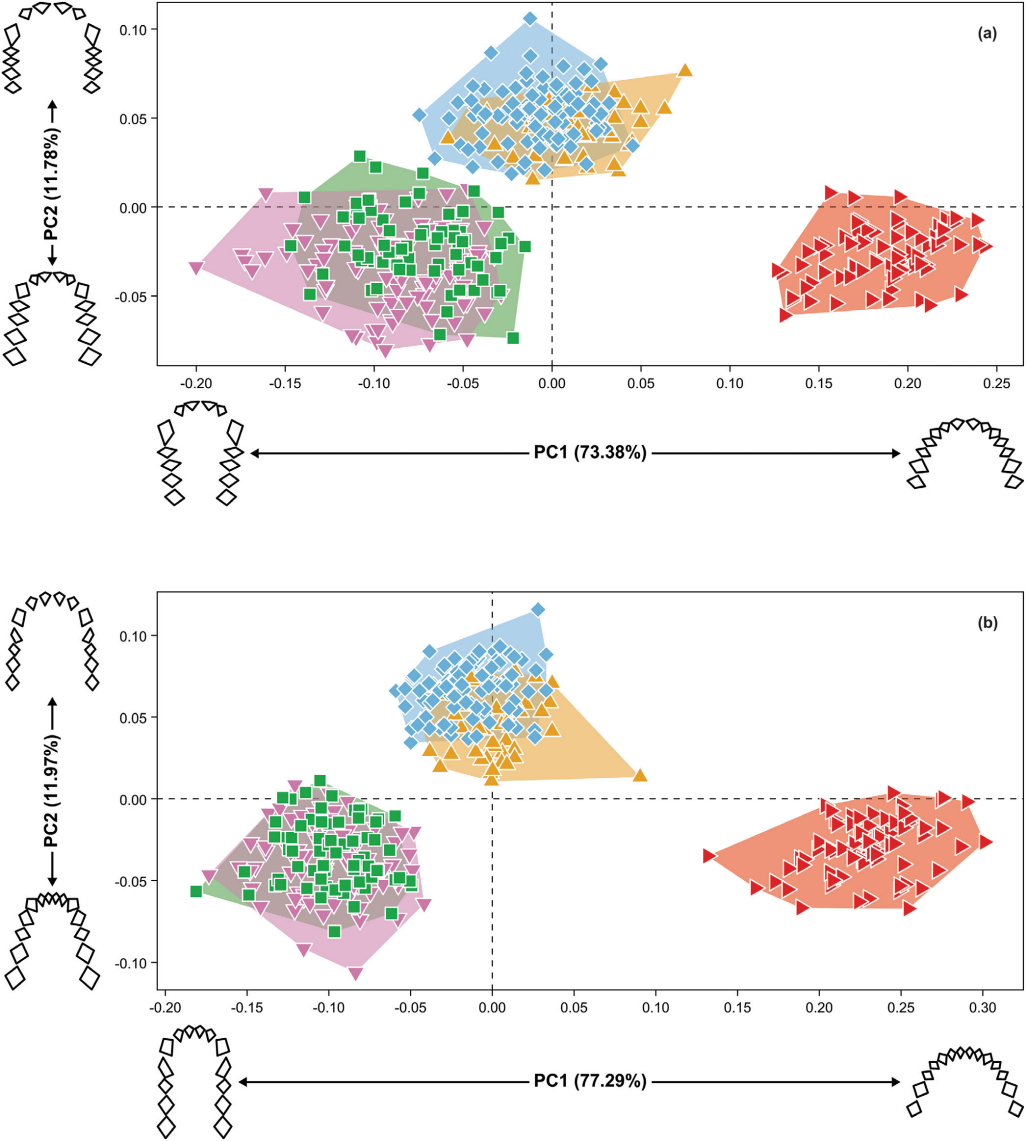
Plots of PC1 and PC2 showing maxillary (a) and mandibular (b) dental arcade shapes of *H. sapiens* (red sideways triangles), *P. troglodytes* (blue diamonds), *P. paniscus* (yellow upwards triangles), *G. gorilla* (green squares), and *G. beringei* (pink downwards triangles). Wireframes show shape differences along PC1 and PC2 in occlusal view.

For both the maxillary and mandibular shape differences (Figure 3), PC1 explains about three‐quarters of the total shape variance and separates *H. sapiens* from the African apes on the positive end, with *Gorilla* having more negative scores than *Pan*. In both jaws, the negative end represents a relatively narrow and U‐shaped dental arcade with relatively long posterior tooth rows and relatively large canine alveoli, while the positive end represents a relatively wide and short parabolic dental arcade. For both the maxillary and mandibular dental arcade, PC2 explains about one‐tenth of the total shape variance and separates *Pan* on the positive end from *Gorilla* and *H. sapiens* on the negative end. In both jaws, the dental arcade is relatively narrow and slightly V‐shaped with relatively smaller incisor alveoli and relatively larger postcanine alveoli at the negative end, compared to relatively wide and U‐shaped dental arcades with relatively larger incisor alveoli and relatively smaller postcanine alveoli at the positive end.

The two‐block partial least squares analysis between the maxillary and mandibular dental arcade shapes shows that all specimens scatter around the diagonal, indicating that the pattern of covariation is similar for all five extant hominine species (Figure S30). Shape differences and species separation along PLS1 and PLS2 (Figure S30) are comparable with those along PC1 and PC2 (Figure 3), respectively. PLS1 explains about 98% of the total shape covariance, with *G. beringei* showing the weakest correlation, followed by *P. troglodytes*, *H. sapiens*, *G. gorilla*, and *P. paniscus* (Table S6). These different magnitudes of covariation do not reflect the conserved shape gradient of the dental arcade on PLS1 (Figure S30a) and thus how tightly the upper and lower jaws covary might be more taxon‐specific than shape‐specific. PLS2 explains only 2% of the total shape covariance, with *P. paniscus* and *H. sapiens* showing lower correlation between their maxillary and mandibular dental arcades than *P. troglodytes*, and the *Gorilla* species showing the strongest correlation (Table S6). These different magnitudes of covariation again do not align with the conserved shape gradient on PLS2 (Figure S30b), suggesting that features like incisor alveolar size and posterior tooth row length are only of minor importance to overall covariation.

### 3.2 Regression models and prediction error (aim 2)

Predicted maxillary dental arcade sizes are both larger and smaller than the original sizes of extant hominine specimens (Figure S31). The mean predicted size is somewhat larger in *P. troglodytes*, *P. paniscus*, and *G. beringei*, but slightly smaller in *H. sapiens* and *G. gorilla*, although differences are only minimal and not significant for *G. beringei* (Table S7). Moreover, these differences do not reflect the size gradient of the extant species, with *P. paniscus* having on average the smallest dental arcades and *G. beringei* the largest, yet in both cases the mean predicted size is slightly increased. Degrees of size variation in the predicted maxillary dental arcades do not significantly differ from those in the original samples for the extant hominine species (Table S8), indicating that including predictions in the fossil sample will not likely bias size variation results.

**FIGURE 4:**
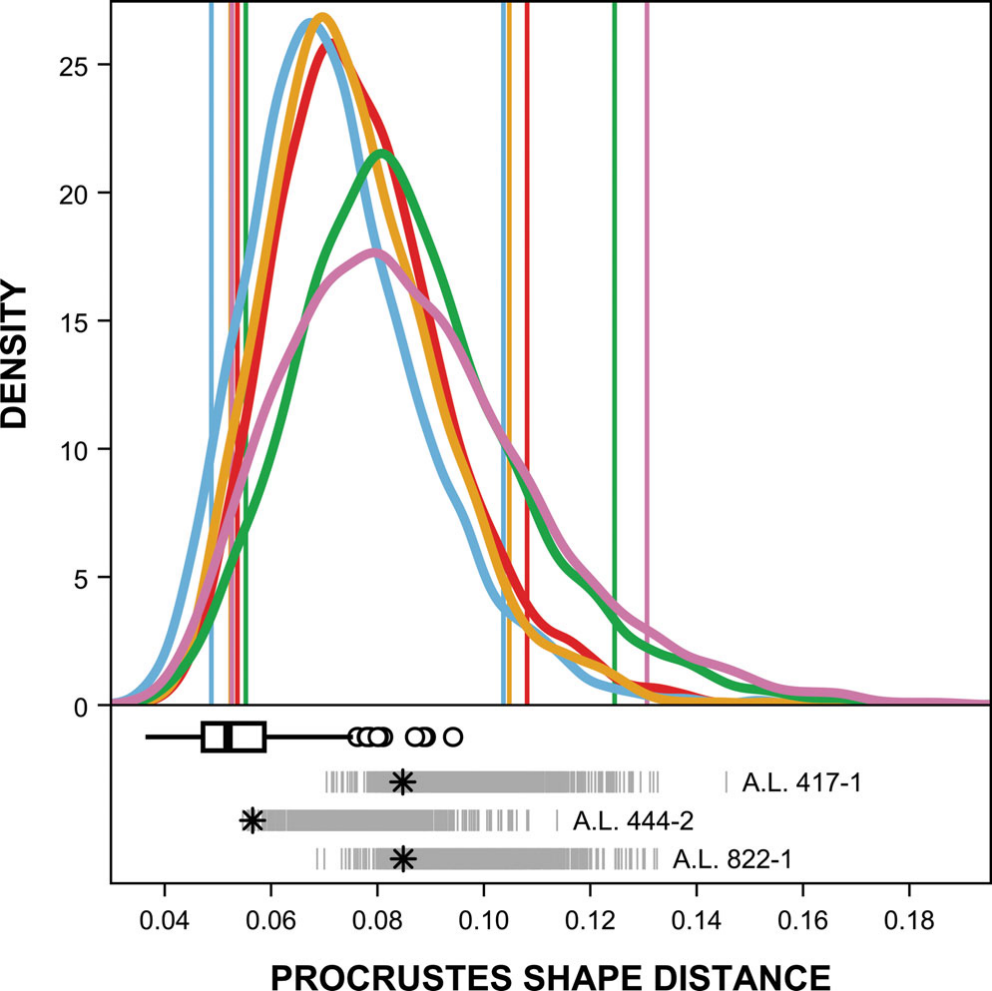
Horizontal boxplot of the Procrustes shape distances between original and predicted maxillary dental arcades for the extant hominine sample, in the context of density plots of the Procrustes shape distances between all possible specimen pairs of the original maxillary dental arcades for *H. sapiens* (red), *P. troglodytes* (blue), *P. paniscus* (yellow), *G. gorilla* (green), and *G. beringei* (pink). Vertical lines represent the 5% and 95% limits of these intraspecific shape distributions. Black stars indicate the Procrustes shape distances between the original maxillary dental arcades and mean corrected predictions of three *A. afarensis* specimens with associated upper and lower jaws. Gray lines indicate prediction uncertainty, showing the Procrustes shape distances between the three original dental arcades and their 1000 corrected predictions.

Figure S32 shows the PC plot for the original maxillary dental arcade shapes and their predictions connected by arrows. Shape differences between original and predicted dental arcades are much smaller than the shape variation within extant hominine species (Figure 4). Hence, the shape of the maxillary dental arcade can be predicted with high accuracy from mandibular landmarks. Even the outliers with the largest prediction error fall within the range of intraspecific shape variation. Moreover, the average Procrustes shape distance between original and predicted dental arcades is smaller for each extant species than the 5% limit of its intraspecific distribution (Table S9).

Extant specimens with the largest shape difference between their original and predicted maxillary dental arcades show relatively small errors (Figure S33). In *H. sapiens*, the largest prediction error concerns an increased length and decreased width of the dental arcade. The positions of the C, P^3^, and M^2^ alveoli somewhat deviate in the predicted maxillary dental arcade of *P. troglodytes* with the largest error, while that of *P. paniscus* has slightly smaller I^1^, I^2^, and M^2^ alveoli with a slightly shifted position. In *Gorilla*, the largest prediction error concerns a less anterior position of the I^1^ and I^2^ alveoli, an altered shape of the C alveoli, and an increased width of the posterior dental arcade due to a buccal shift of the postcanine alveoli.

The degree of shape variation is significantly decreased in predicted maxillary dental arcades for all extant hominine species (Table S10). The fitted values contain only about half of the information about variation, meaning that the residual errors contain the remainder. This reduced shape variation in predicted dental arcades is also illustrated in Figure S32 as most of the arrows showing the difference between original and predicted shapes point inwards of each extant hominine species' convex hull.

### 3.3 Fossil predictions (aim 3)

The predicted maxillary size of A.L. 417‐1 is somewhat smaller than the original, while those of A.L. 444‐2 and A.L. 822‐1 are slightly larger (Figure S31). Procrustes shape distances between the original maxillary dental arcades and mean corrected predictions for these three *A. afarensis* specimens fall within the range of intraspecific shape variation of the extant hominine species, yet corrected predictions can be far less accurate as shown by the prediction uncertainty range (Figure 4). Predictions are most accurate for A.L. 444‐2, while those for A.L. 417‐1 and A.L. 822‐1 differ mainly in the shape and position of the anterior dentition, especially the I^2^ and C, resulting in somewhat more U‐shaped rather than parabolic dental arcades (Figure S34). Note that the Procrustes distance between the original shape and mean corrected prediction is similar to that between the original shape and uncorrected fitted values for these three *A. afarensis* specimens. While corrections do not improve prediction accuracy at the specimen level, they do account for potential variation at the species level that is not captured by the fitted values.

**FIGURE 5:**
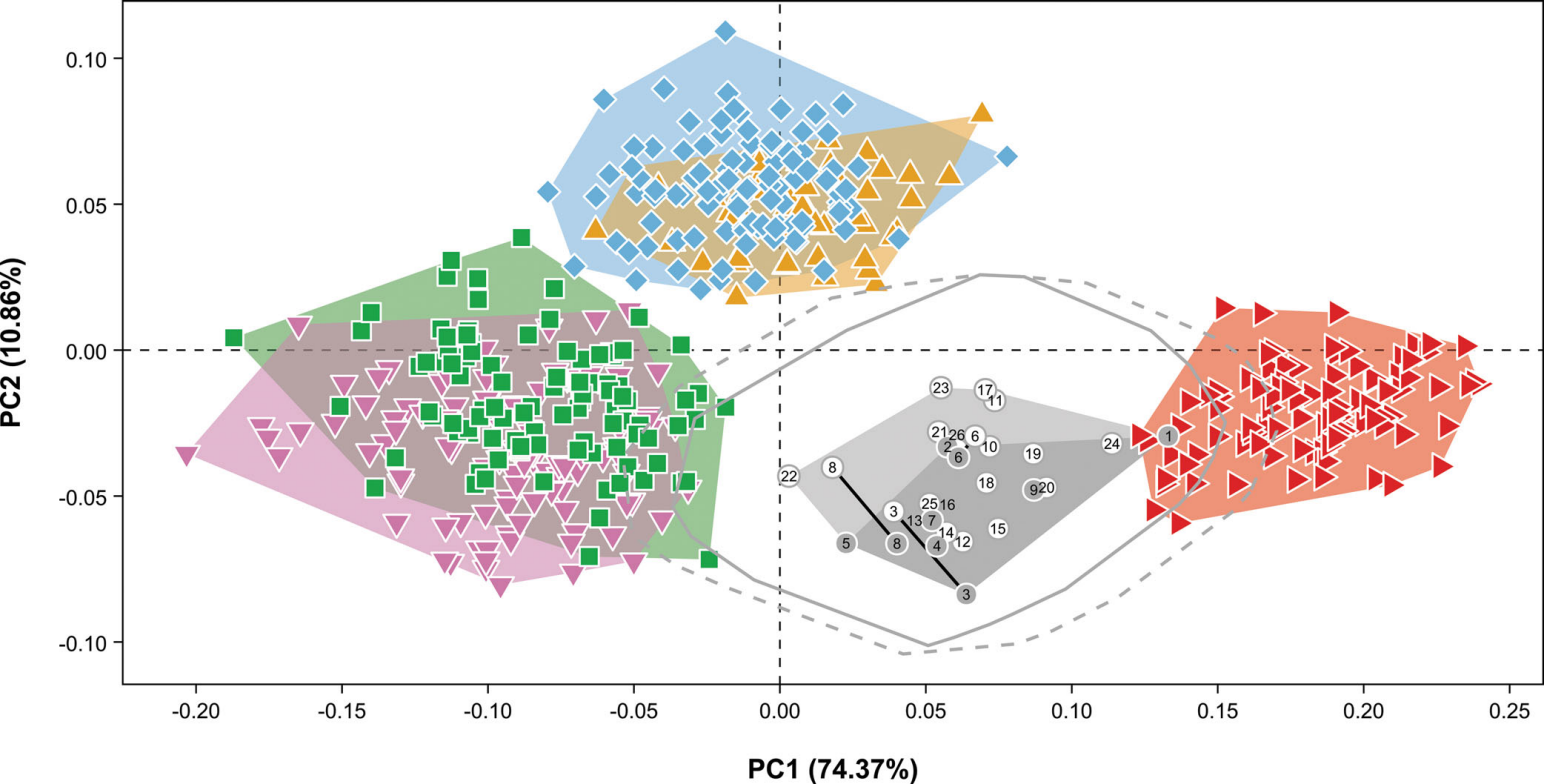
Plot of PC1 and PC2 showing maxillary dental arcade shapes of *A. afarensis* (gray circles), *H. sapiens* (red sideways triangles), *P. troglodytes* (blue diamonds), *P. paniscus* (yellow upwards triangles), *G. gorilla* (green squares), and *G. beringei* (pink downwards triangles). For *A. afarensis*, the gray circles represent actual maxillae and the white circles the mean corrected predictions obtained from *A. afarensis* mandibular landmarks. Lines connect the original maxillary dental arcade shapes and mean corrected predictions of three *A. afarensis* specimens with associated upper and lower jaws. Prediction uncertainty is indicated by the 1sd (solid gray line) and 2sd (dashed gray line) convex hulls. See Table 1 for *A. afarensis* specimen numbers.

Predicted fossil dental arcades occupy part of the shape space not observed in the original *A. afarensis* maxillary sample, including more negative PC1 scores that represent relatively narrower and somewhat more U‐shaped arcades and more positive PC2 scores that represent dental arcades with relatively larger incisor alveoli (Figure 5). Moreover, the 1sd and 2sd convex hulls show the prediction uncertainty and thus where in shape space predicted *A. afarensis* specimens could potentially fall with 68% and 95% confidence, respectively. These convex hulls do not necessarily indicate the full range of potential shape variation, as the filled gray convex hull showing the possible extent of the *A. afarensis* distribution can move within the 1sd and 2sd convex hulls but will not necessarily take up the entire space within. Still, the true convex hull for *A. afarensis* is likely larger than the gray convex hull currently shown as variation is reduced in the predictions.

### 3.4 Size and shape variation (aim 4)

Maxillary dental arcades of *A. afarensis*, *G. gorilla*, and *G. beringei* are significantly more variable in size than those of *H. sapiens*, *P. troglodytes*, and *P. paniscus* (Table S11). Including predicted sizes in the *A. afarensis* sample does not alter these results.

**FIGURE 6:**
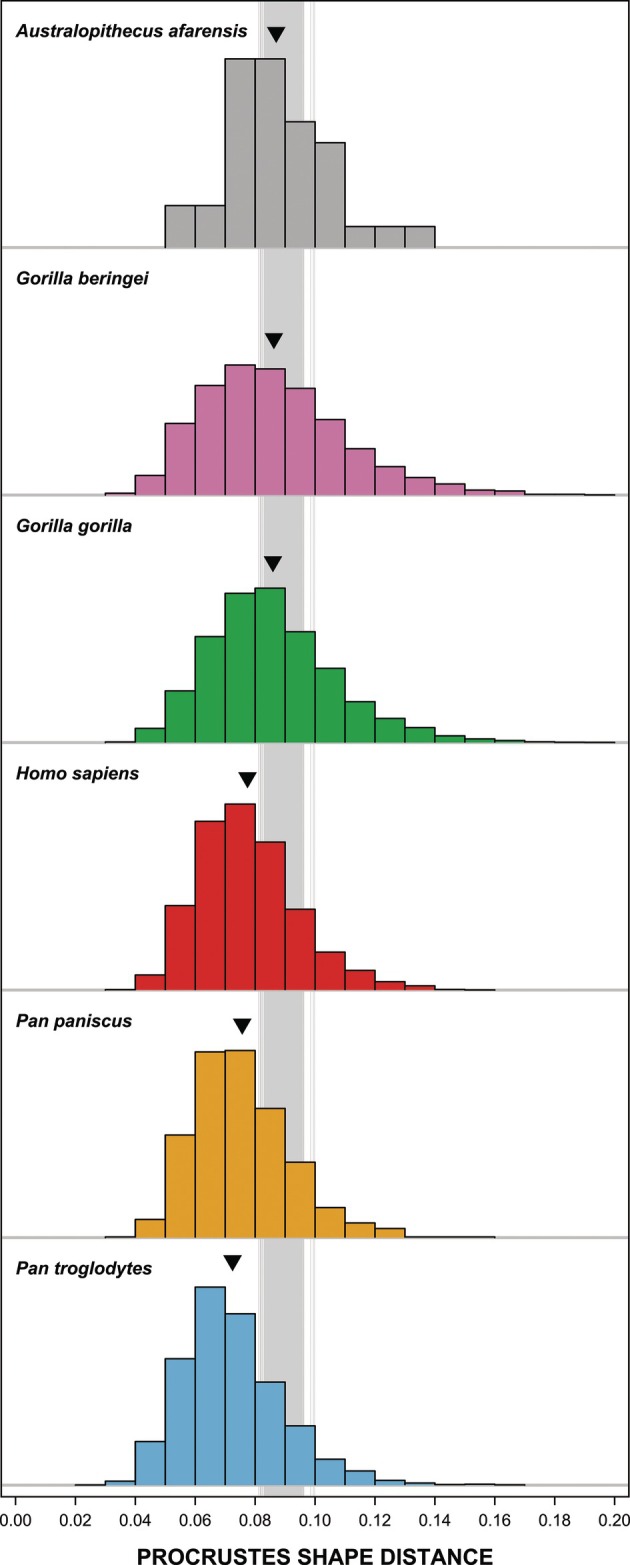
Density histograms of the Procrustes shape distances between all possible specimen pairs for each species when analyzing original maxillary dental arcades only, ordered from smallest to largest mean value (black arrow). Colors as in Figure 5. Gray lines show mean values when including randomly sampled corrected predictions in the *A. afarensis* sample.

The degree of shape variation is highest in *A. afarensis* and the *Gorilla* species, and then significantly decreases from *H. sapiens* to *P. paniscus* to *P. troglodytes* (Figure 6 and Table S12). When analyzing randomly sampled corrected predictions together with the original *A. afarensis* maxillary dental arcades, the mean Procrustes shape distance between all possible specimen pairs ranges between 0.08115 and 0.09982 (Figure 6) and there is about a 50%–60% probability that the degree of shape variation is significantly higher compared to *G. gorilla* and *G. beringei* (Table S13).

In *H. sapiens*, continent of origin explains 12% of the total shape variance (Table S14). Sex explains around 20% of the total shape variance in the *Gorilla* species and also has a significant effect in *P. troglodytes* and *P. paniscus*, but differences between males and females only explain 8% and 10% of the total shape variance, respectively. Sexual dimorphism does not significantly affect the dental arcade shape of *H. sapiens*.

### 3.5 Static allometry (aim 5)

Size has a significant effect on the dental arcade shapes of all extant hominine species, with the explained variance ranging between 3% and 20% (Table S15). The fraction of shape variance explained by size is 7% for the original *A. afarensis* maxillary dental arcades, while it ranges between 1% and 10% when including randomly sampled corrected predictions (Table S16). Only in a few random samples is the size–shape regression significant for *A. afarensis*, but the probability of this occurring is extremely low, with about 98% of the analyses being not significant.

Although the effects of size (Table S15) and sex (Table S14) on dental arcade shape are somewhat similar for the extant hominine species, in most cases, except for *P. paniscus*, the multivariate regression model that best explains shape includes the interaction between size and sex (Table S17), supporting the interpretation that both factors contribute independently and interactively to shape variation. In *P. paniscus*, however, size and sex both have an effect on shape; yet this is not cumulative.

### 3.6 Temporal trends (aim 6)

**FIGURE 7:**
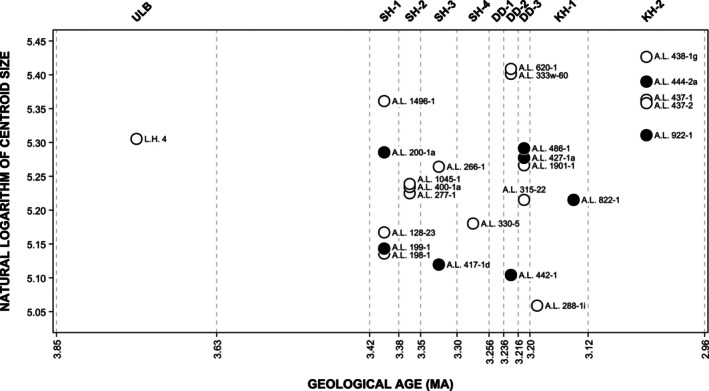
Temporal trends in the natural logarithms of centroid sizes, based on the placement of *A. afarensis* specimens in the stratigraphic units of Laetoli and Hadar. Original maxillary dental arcades are shown as filled black circles and predictions as open black circles.

Figure 7 plots the natural logarithms of centroid sizes of the *A. afarensis* specimens against their placement in the stratigraphic units of Laetoli and Hadar. Maxillary dental arcade size does not change significantly over time (*r* = 0.324, *t* = 1.678, *p* = 0.106). Additionally, maxillary dental arcade shapes do not show a clear directional change over time in *A. afarensis* (Figure S35).

### 3.7 Sexual dimorphism (aim 7)

Sex assignments of *A. afarensis* specimens obtained from previous studies (Table 1) are presented in the context of size and stratigraphic units in Figure S36. Two *A. afarensis* mandibles have no sex assignment in the literature, and their sex was therefore estimated from overall dental arcade sizes (Figure S37), absolute labiolingual widths of the canine alveoli (Figure S38), and absolute buccolingual widths of the third premolar (Figure S39) and second molar (Figure S40) alveoli. A.L. 1045‐1 is intermediate in dental arcade size but has one of the narrowest C¯ and M_2_ alveoli, and its P_3_ alveolar width falls within the female range. This specimen was therefore classified as female. A.L. 1901‐1 also has an intermediate dental arcade size, but given that its C¯ and P_3_ alveolar widths are high within the male range of variation and its M_2_ alveolus is the widest of the mandibular sample, this specimen was classified as male.

**FIGURE 8:**
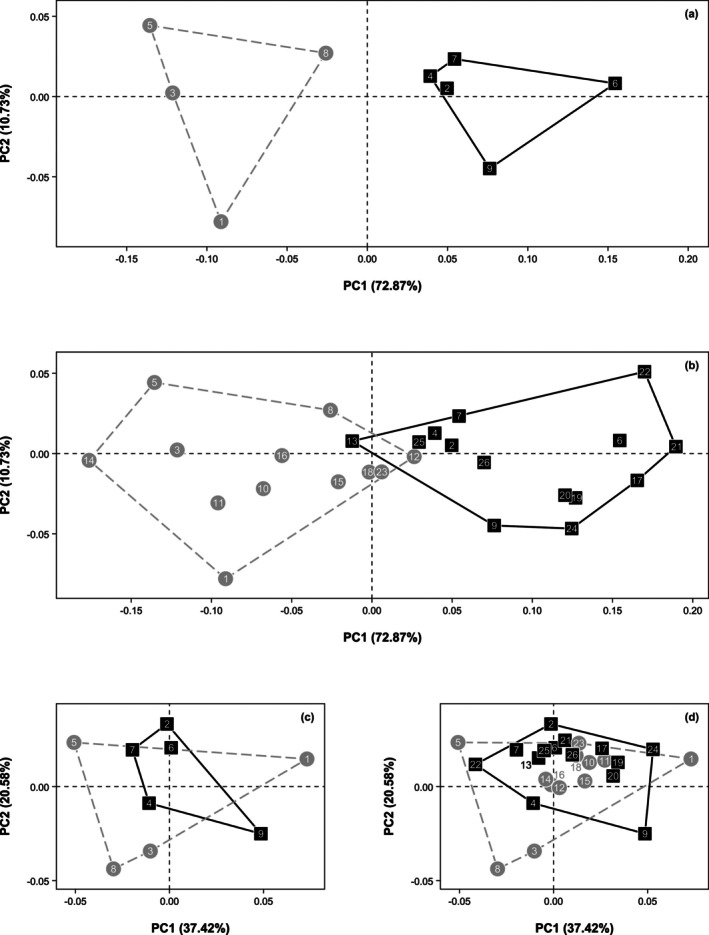
Sexual dimorphism trends between the maxillary dental arcades of *A. afarensis* males (black squares) and females (gray circles) on PC1 and PC2 in form (a, b) and shape (c, d) space. Data is shown for the original maxillary dental arcade shapes (a, c) and including the mean corrected predictions (b, d). See Table 1 for *A. afarensis* specimen numbers.

For all species, males have significantly larger dental arcades than females, especially *A. afarensis* and *Gorilla*, as indicated by the male/female ratios (Table S18). Given that one of the main criteria used in the sex determination of fossils is size, the large male/female ratio for *A. afarensis* is not surprising. In both form and shape space, males and females are not well separated for *H*. sapiens, *P. troglodytes*, and *P. paniscus*, while there is little overlap between G. *gorilla* and *G. beringei* sexes (Figure S41). *Australopithecus afarensis* males and females overlap considerably in shape space, but are separated in form space, although less so when including predicted dental arcades (Figure 8).

**FIGURE 9:**
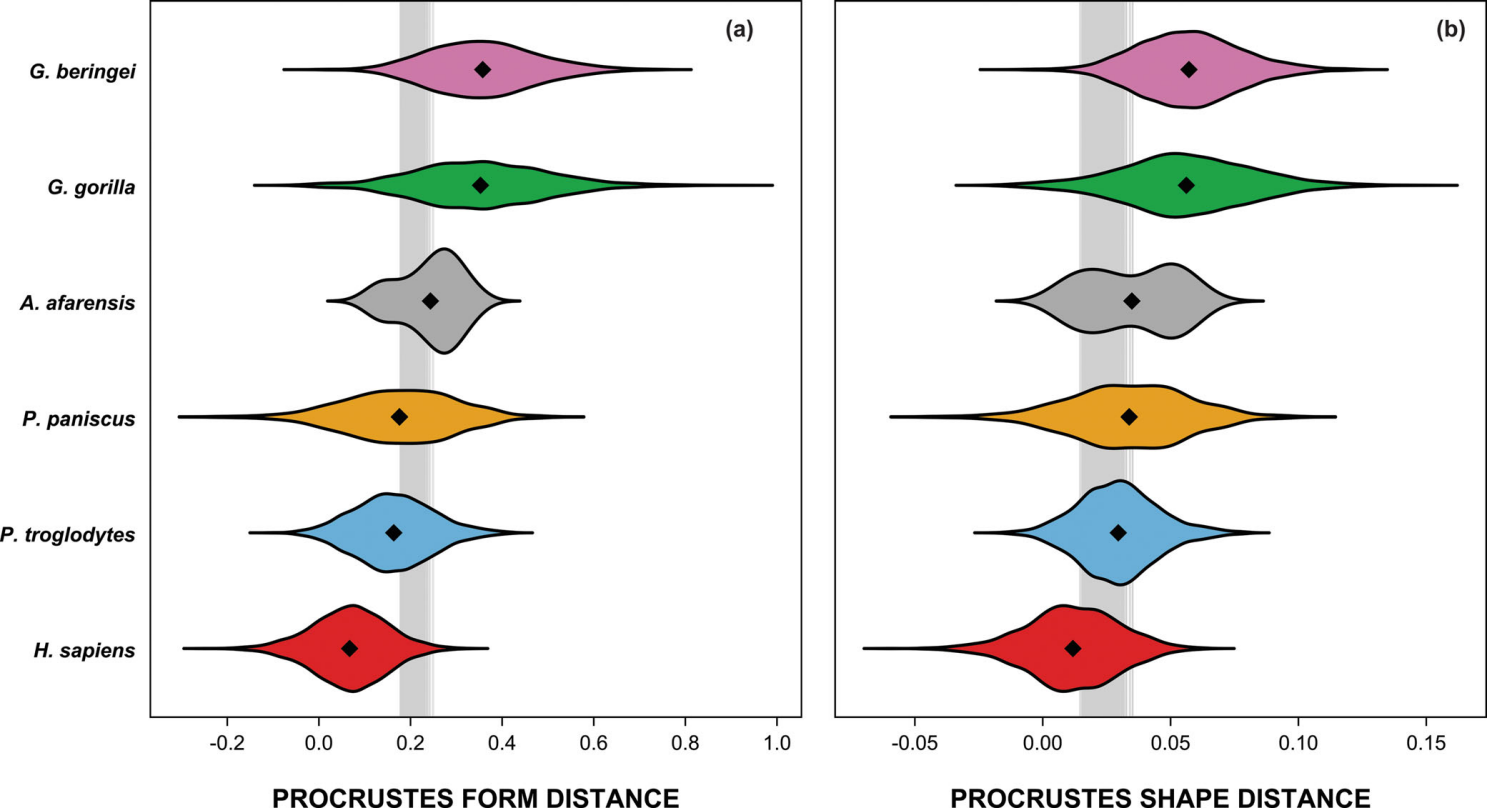
Distribution plots of the geometrically manipulated Procrustes form (a) and shape (b) distances between all possible pairs of males and females for each species when analyzing original maxillary dental arcades only, ordered from smallest to largest mean value (black diamond). Colors as in Figure 5. Gray lines show mean values when including randomly sampled corrected predictions in the *A. afarensis* sample.

The degree of sexual form dimorphism significantly decreases from the *Gorilla* species to *A. afarensis* to *P. paniscus* to *P. troglodytes* to *H. sapiens* (Figure 9a and Table S19). In shape space, differences between *A. afarensis* males and females are not significantly different from those in the species of *Pan* (Figure 9b and Table S19). When analyzing randomly sampled corrected predictions together with the original *A. afarensis* maxillary dental arcades, the degree of sexual dimorphism is reduced (Figure 9). The mean Procrustes form and shape distance between all possible pairs of males and females ranges between 0.17678 and 0.25045 and between 0.01443 and 0.03537, respectively. Form differences between males and females in the extended *A. afarensis* sample are less likely to be significantly different from those in *P. paniscus* (Table S20). In shape space, the degree of sexual dimorphism in *A. afarensis* is most likely significantly lower than in the *Pan* species when including predictions.

**FIGURE 10:**
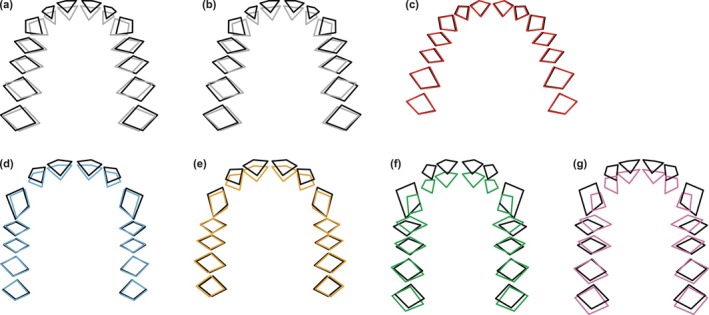
Differences between mean male (black) and female (color) maxillary dental arcade forms on PC1 and PC2 for *A. afarensis* (a, b; gray), *H. sapiens* (c; red), *P. troglodytes* (d; blue), *P. paniscus* (e; yellow), *G. gorilla* (f; green), and *G. beringei* (g; pink), shown in occlusal view. For *A. afarensis*, data is shown for the original maxillary dental arcade shapes (a) and including the mean corrected predictions (b). Forms are rotated to minimize the distance between postcanine alveolar landmarks and differences are magnified twice for better visualization.

**FIGURE 11:**
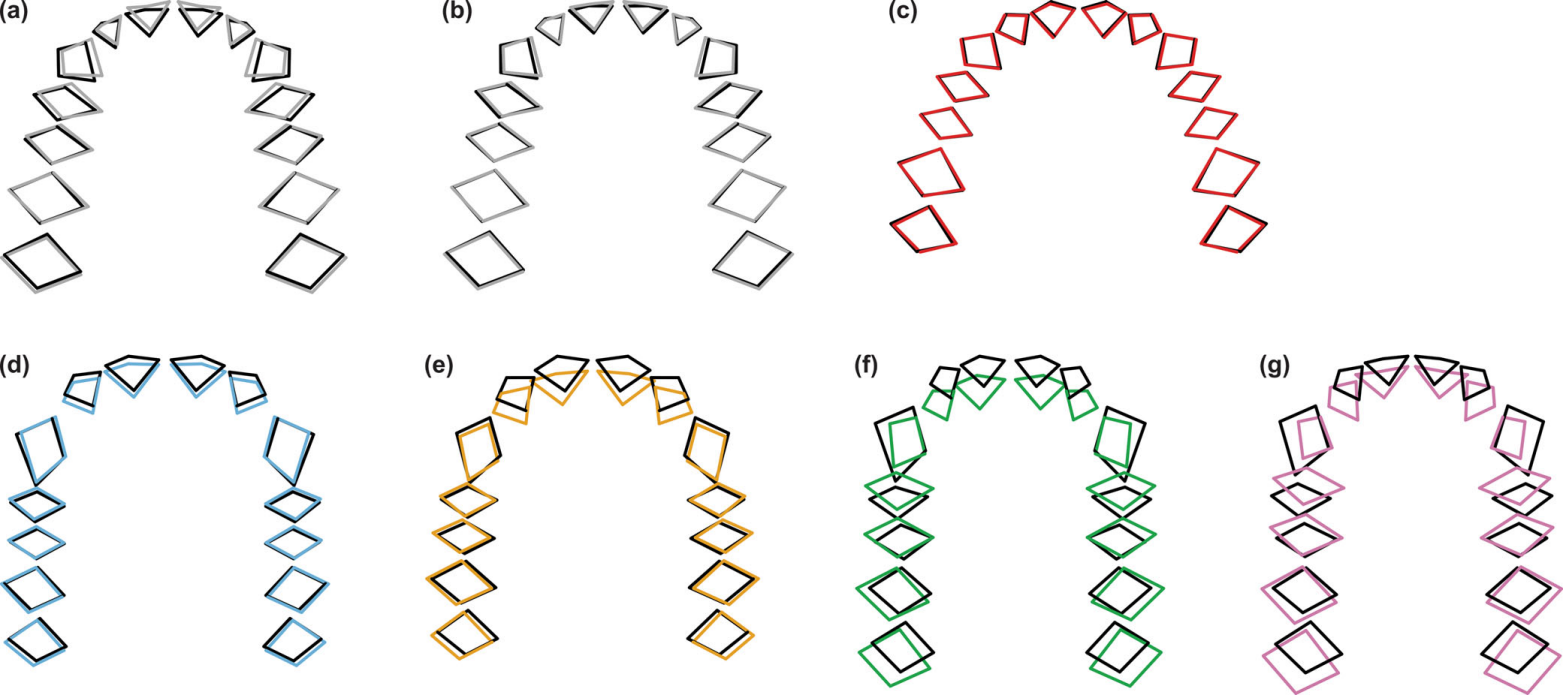
Differences between mean male (black) and female (color) maxillary dental arcade shapes on PC1 and PC2 for *A. afarensis* (a, b; gray), *H. sapiens* (c; red), *P. troglodytes* (d; blue), *P. paniscus* (e; yellow), *G. gorilla* (f; green), and *G. beringei* (g; pink), shown in occlusal view. For *A. afarensis*, data is shown for the original maxillary dental arcade shapes (a) and including the mean corrected predictions (b). Shapes are rotated to minimize the distance between postcanine alveolar landmarks and differences are magnified twice for better visualization.

Patterns of sexual dimorphism between *A. afarensis* males and females are different from those between *H. sapiens*, *Pan*, and *Gorilla* sexes. In form space, dental arcades of *A*. *afarensis* males project more anteriorly, have slightly larger I^2^/C interalveolar distances, and are somewhat wider compared to those of females (Figure 10a,b). Maxillary dental arcades of *H. sapiens* males and females show no differences (Figure 10c). African ape males have to a variable degree more anteriorly projecting dental arcades with larger C alveoli and I^2^/C interalveolar distances than females (Figure 10d–g). These differences are more pronounced in *Gorilla* than in *Pan*. Moreover, *Gorilla* males also have somewhat shorter posterior tooth rows (Figure 10f,g). In shape space, the I^1^ alveoli of *A. afarensis* males have somewhat less relative anterior projection and the I^2^/C interalveolar distances are larger than in females when only analyzing original maxillae (Figure 11a), while no differences are observed in dental arcade shape between the *A. afarensis* sexes when including mean corrected predictions (Figure 11b). Likewise, *H. sapiens* sexes do not differ in their dental arcade shapes (Figure 11c). To a variable degree, dental arcades of African ape males project relatively more anteriorly, with relatively larger C alveoli and I^2^/C interalveolar distances, but relatively shorter posterior tooth rows compared with females (Figure 11d–g). Again, these differences are more marked in *Gorilla* compared with *Pan*.

### 3.8 Late juveniles (aim 8)

The *A. afarensis* late juveniles included in this study fall within the adult range of size variation (Figure 7). A.L. 128‐23 is comparable in size to the adults A.L. 198‐1 and A.L. 199‐1 from the SH‐1 submember, and A.L. 486‐1 has a similar size as the adults A.L. 427‐1a and A.L. 1901‐1 from the DD‐3 submember. Moreover, A.L. 128‐23 and A.L. 486‐1 fall within the adult range of variation on PC1 and PC2 for the mandibular and maxillary dental arcade shapes, respectively (Figure S25), and their dental arcade shapes are similar to the mean for adults (Figure S42).

## 4 DISCUSSION

In this study, the maxillary dental arcade morphology of *A. afarensis* and extant hominine species was examined, integrating evidence obtained from mandibles and thereby increasing the fossil sample size from nine to 26. The main findings are that (1) degrees of size and shape variation are high in *A. afarensis* in the context of extant hominine variation, potentially even higher than in *Gorilla* species when including predictions in the fossil sample, (2) no allometry was detected, even when expanding the *A. afarensis* sample with predictions, (3) size and shape do not significantly change over time when analyzing original and predicted *A. afarensis* dental arcades together, and (4) sexual form and shape dimorphism are reduced when including *A. afarensis* predictions in the fossil sample, although sexual size differences remain similar.

### 4.1 Prediction accuracy

The covariation between maxillary and mandibular dental arcades was explored for modern humans and African apes. High levels of covariation were found, which are consistent with results for previously studied extant hominoid samples (Spoor et al., 2015; Stelzer et al., 2017). Although the different magnitudes of shape covariation do not reflect the conserved shape gradient of the dental arcade on PLS1 or PLS2, it is noteworthy that the two species with the weakest correlation on PLS2 are also the least dimorphic, suggesting that sexual dimorphism in canine size might potentially influence the magnitude of covariation on this axis. In species with pronounced canine dimorphism, the need for functional occlusion of canines may impose stronger morphological integration between the upper and lower jaws. Conversely, in species with reduced dimorphism, the relaxed constraints on canine fit could allow for more independent variation in jaw shape, resulting in a weaker correlation on PLS2. This pattern raises an important consideration regarding *H. sapiens*, which exhibits the most extreme reduction in canine dimorphism among the taxa studied. While this reduction might contribute to decreased covariation on PLS2, it is likely not the sole driving factor, which may explain why *H. sapiens* is not an outlier in this context.

That all datapoints scatter along a diagonal for both size and shape suggests a common pattern of covariation in the dental arcades of extant hominine species. Evolutionary changes leading to differences between these taxa are thus not associated with alterations to the underlying covariation of the upper and lower dental arcade. This also strengthens our assumption that fossil hominin jaws likely exhibit a similar pattern of covariation to those of extant hominine species.

In this context, maxillary dental arcades were predicted from mandibular landmarks, and the accuracy of predictions was tested for the extant hominine dataset and three *A. afarensis* specimens with associated upper and lower jaws (A.L. 417‐1, A.L. 444‐2, and A.L. 822‐1). Both the size and shape of the maxillary arcade can be predicted with high accuracy, although shape variation significantly decreases in the fitted values (Table S10). A novel method was developed to correct for this reduced shape variation, adding randomly sampled residual errors to the fitted predictions. These corrected predictions show the extent of the prediction uncertainty for the fossil sample, noting that shape differences between original maxillary dental arcades and corrected predictions for the three aforementioned *A. afarensis* fossils never fall outside the range of intraspecific shape variation of the extant hominine species (Figure 4). Prediction error in these specimens mostly concerns the shape and position of the anterior teeth, corroborating previous studies that found this area to be more prone to prediction inaccuracies than the postcanine dentition (Spoor et al., 2015; Stelzer et al., 2018). Furthermore, the inclusion of late juveniles in the fossil sample is not expected to have a major impact given that both A.L. 128‐23 and A.L. 486‐1 fall within the size and shape variation of the *A. afarensis* adults.

### 4.2 Impact of restricted morphology

Since predictions are restricted to the maxillary alveolar process and information about the midplane palate, subnasal area, nasal aperture, zygomatic root, and lower orbital margin is lost as a consequence, it is of particular interest to assess the implications of only examining the dental arcade instead of the entire maxillary morphology. Conclusions about size remain similar, except that sexual size dimorphism is significant for *P. paniscus* when only assessing the dental arcade (Table S18), but not when analyzing entire maxillae (Hanegraef & Spoor, 2025, table S11). However, the statistical significance of these size differences between *P. paniscus* males and females is, in both cases, close to the *p* < 0.05 threshold.

The *H. sapiens* maxilla has the most variable shape of the sample (Hanegraef & Spoor, 2025, table S8), while the degree of shape variation of its dental arcade is significantly lower than in *A. afarensis* and the *Gorilla* species (Table S12). A potential explanation is that geographical distribution and sexual dimorphism affect the maxillary alveolar process of *H. sapiens* less than the rest of its maxillary shape, and the explained variance of both factors indeed suggests such a trend (Table S14) (Hanegraef & Spoor, 2025, table S9). The body of the maxilla partly reflects nasal cavity size and shape, which is known to be affected by geography and climate (Noback et al., 2011). Moreover, *H. sapiens* males and females were observed to differ mainly in maxillary height and not in their dental arcade shape (Hanegraef & Spoor, 2025, fig. 13e–h).

No significant difference was detected in the degree of maxillary shape variation for the *Pan* species (Hanegraef & Spoor, 2025, table S8), yet *P. paniscus* has a more variable dental arcade shape than *P. troglodytes* (Table S12). In contrast, the degrees of maxillary shape variation and sexual form and shape dimorphism were observed to be significantly different between *G. beringei* and *G. gorilla* (Hanegraef & Spoor, 2025, tables S8 and S12, respectively), while no significant differences were found here for their dental arcade shapes (Tables S12 and S18, respectively). These trends show that shape variation can vary even between closely related taxa depending on which morphological features are included in the analysis. Similarly, although sex does not need to be considered when adjusting for allometry in the entire maxilla (Hanegraef et al., 2022), the multivariate shape regression model comparisons in this study indicate sex should be taken into account for allometric adjustments of the maxillary dental arcade in most extant hominine species, except for *P. paniscus*.

The degree of sexual shape dimorphism reduces in *A. afarensis* from a level between *G. gorilla* and *P. paniscus* for the maxilla (Hanegraef & Spoor, 2025, table S12) to a level similar to the *Pan* species when only assessing the dental arcade (Table S19). As with *H. sapiens*, males and females of *A. afarensis* mainly differ in maxillary height (fig. 13a–d in Hanegraef & Spoor, 2025), potentially explaining the lower level of sexual dimorphism in their dental arcade shapes.

### 4.3 Impact of increased fossil sample

Mandibular evidence is integrated here to assess the maxillary dental arcade morphology and hence it is of interest to discuss the consequences of increasing the fossil sample size from nine to 26. Compared to analyses of original *A. afarensis* maxillary dental arcades, including predicted data in the fossil sample does not impact the degree of size variation and still no allometric trend can be detected. The low probability of a significant size effect in *A. afarensis* is likely linked to the relatively small sample size for this species compared to those for the extant hominine taxa. Moreover, the wide range of shape variance explained by size when including randomly sampled corrected predictions (Table S16) highlights the strong influence of sample composition on the observed size‐shape relationship. Hence, potentially even more specimens are needed to confidently analyze intraspecific static allometry in *A. afarensis*.

**FIGURE 12:**
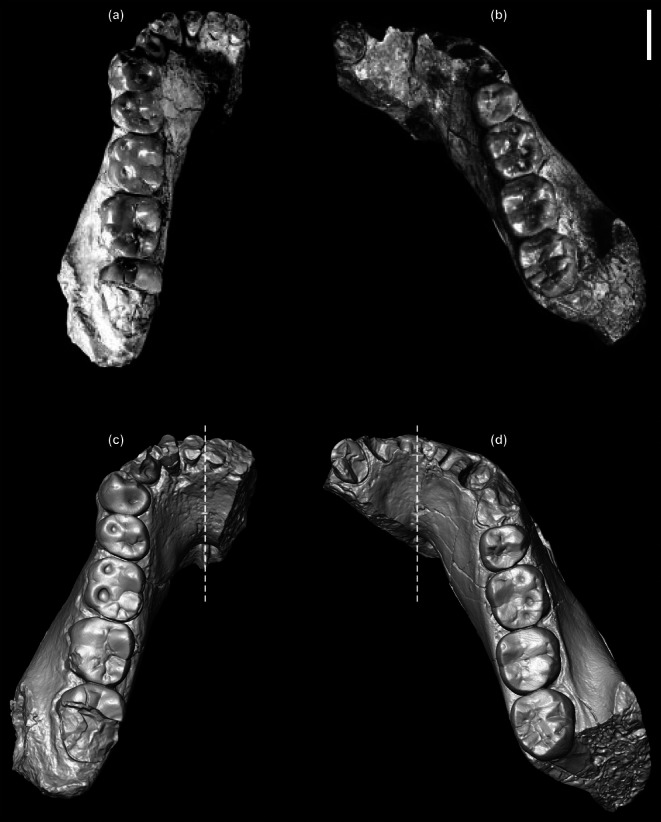
Occlusal view of the *A. afarensis* mandibles A.L. 417‐1a (a, c) and A.L. 330‐5 (b, d). Photographs (a, b) show how the specimens are compared in Kimbel and Delezene (2009), fig. 14) to demonstrate variation in dental arch shape. The 3D surface visualizations (c, d) realign these specimens based on the correct orientation of their midplane (dotted line). Scale bar 1 cm.

The degree of shape variation in the extended *A. afarensis* sample is often higher than observed in the nine original specimens, with about a 50%–60% probability that the intraspecific shape variation is even larger than in *G. gorilla* and *G. beringei*, the most variable species of the extant hominines. High variability in *A. afarensis* of dental arcade divergence and mandibular corpus breadth has been noted before (Kimbel & Delezene, 2009; Kimbel & White, 1988; Kimbel et al., 2004; Lockwood et al., 2000). Now the use of previously unavailable evidence from μCT and 3D virtual reconstruction makes a particularly beneficial contribution in this context because the visual appearance of a fossil as preserved can give an incorrect impression of the original shape (Figure 12).

When teeth are preserved but the alveolar margin is eroded or completely absent, particularly common in the anterior dentition, there is some uncertainty regarding the inferosuperior position of landmarks. Although such estimations are unavoidable, they could potentially contribute to an increase in the degree of shape variation. To assess the impact of this uncertainty, intraspecific shape variation was reanalyzed using just 2D data by projecting the 3D Procrustes coordinates onto the occlusal plane defined for each specimen by their mesial P^3^ and distal M^2^ landmarks. When examining the original *A. afarensis* maxillae only, results for the 2D (Table S21) and 3D (Table S12) analyses are comparable, with the degree of shape variation in *A. afarensis* not being significantly different from those of *Gorilla* species. However, when predicted *A. afarensis* maxillary dental arcade shapes were included, the 2D analysis indicates a 58% probability that the degree of variation is statistically similar to *Gorilla* (Table S22), while the 3D analysis showed a 50–60% probability of statistically higher variation compared with *G. gorilla* and *G. beringei* (Table S13). This result suggests that variation along the inferosuperior axis does contribute to the overall shape variation observed in *A. afarensis*, though removal of this component still results in a degree of intraspecific variation as high as that observed in *Gorilla*. Thus, while a 2D analysis slightly reduces the apparent shape variation, the high variation in *A. afarensis* dental arcades persists independently of inferosuperior landmark positioning. In addition, it is worth mentioning that irregular preservation of the labial alveolar margin is also seen in skeletal collections of extant great apes and modern humans. Hence, the higher variability of these landmarks, intrinsic to the local morphology, will unavoidably be present in this type of study in general.

It should also be acknowledged that the substantial shape variation within the *A. afarensis* sample has led some to interpret it as evidence for the presence of multiple species. This perspective has a long‐standing historical precedent, particularly in the 1980s, when several researchers suggested that more than one hominin taxon might be represented within the *A. afarensis* hypodigm (e.g., Falk et al., 1995; Olson, 1981, 1985; Sénut, 1983; Tardieu, 1983). However, subsequent comprehensive analyses have consistently failed to identify reliable criteria for dividing the sample into distinct species (see Kimbel & Delezene, 2009 for a review). In light of these findings, we do not consider our data to support the hypothesis of taxonomic heterogeneity within *A. afarensis*.

No significant temporal size trend is detected when including predicted specimens in the *A. afarensis* sample. It thus seems likely that the size increase after ca. 3.2 Ma detected in Hanegraef and Spoor (2025) for the *A. afarensis* maxillae was indeed a consequence of low sample size. However, when looking at maxillary dental arcade sizes separately for the sexes (Figure S36), males appear to increase in size over time while females decrease somewhat in size, which could explain why no size trend was detected when analyzing both sexes together. Note that the interpretation of these results is complicated as females are absent from both the youngest and oldest temporal groups, with the latter represented by only a single male specimen. Furthermore, our data alludes to a potential increase in size variation over time as differences between the smallest and largest specimens are greater in the youngest temporal groups compared to the oldest, and this would match with the implied divergent size trends for males and females.

In contrast with our findings, Lockwood et al. (2000) and Haile‐Selassie et al. (2016) did observe a significant trend towards larger corpus sizes over time when analyzing a substantial sample of mandibles, including specimens from Maka, Dikika, and Woranso‐Mille, which are not included in our analysis. Both studies found that mainly corpus height and not corpus breadth drives the size increase, and the former dimension is not investigated here. Mandibular corpus height measurements are available for 32 *A. afarensis* specimens from published literature (Table S23) (Alemseged et al., 2005; Haile‐Selassie et al., 2016; Harrison, 2011; Kimbel et al., 2004; Melillo et al., 2021; White, 1977; White et al., 2000; White & Johanson, 1982) and additional data was obtained from CT scans for four Hadar specimens (A.L. 822‐1, A.L. 1045‐1, A.L. 1180‐1, and A.L. 1496‐1). While Lockwood et al. (2000) and Haile‐Selassie et al. (2016) used rank correlations to assess temporal trends, the availability of geological age ranges now enables a more precise analysis of changes in mandibular corpus height over time using Pearson's product–moment correlation tests. Results show a significant increase in mandibular corpus height at M_1_ level (Figure S43a; *r* = 0.476, *t* = 2.868, *p* = 0.008). MAK‐VP‐1/12, NFR‐VP‐1/29, and LDD‐VP‐1/167 align well with Hadar specimens of comparable geological ages, whereas DIK‐2‐1 exhibits a deep mandibular corpus for its geological age, resembling younger specimens such as A.L. 333w‐60 and A.L. 437‐2, as already noted by Alemseged et al. (2005). Moreover, Harrison (2011) remarked on the deep mandibular corpus of Laetoli specimen L.H. 29, which deviates from the observed size trend. Note that the provenance of L.H. 29 has been in question, but regardless, its morphology associates the specimen with *A. afarensis* (Harrison, 2011). Corpus height can only be measured at M_2_ level for L.H. 29, and analyzing all available data at this tooth level reveals no significant temporal change (Figure S43b; *r* = 0.142, *t* = 0.717, *p* = 0.480). This calls into question whether the increase in mandibular corpus height was a more localized phenomenon in the youngest Hadar specimens rather than a species‐wide temporal trend (Harrison, 2011). Furthermore, L.H. 29 highlights that limited sample sizes at the extremes of the *A. afarensis* temporal range, with females absent in both groups, can majorly influence our understanding of temporal trends. Restricting analysis to 3.45–3.12 Ma, a time period with adequate representation of specimens and both sexes, reveals no change in mandibular corpus height at M_1_ level (*r* = 0.055, *t* = 0.266, *p* = 0.793) or M_2_ level (*r* = 0.009, *t* = 0.041, *p* = 0.968). Notably, the bimodal clustering in corpus height at M_1_ level, previously associated with sexual dimorphism (Kimbel et al., 2004), does not appear at the M_2_ level. Moreover, the pattern does not correspond clearly with presumed sex classifications. For example, A.L. 417‐1 and A.L. 822‐1, typically regarded as females, fall within the upper cluster, whereas L.H. 4, often considered a male, falls within the lower cluster.

The degree of sexual size differences remains similar between the original and expanded *A. afarensis* samples as is evident from the male/female ratios (Table S18). In contrast, males and females overlap more in form and shape when including predicted dental arcades. Compared with the nine original maxillary dental arcades, the degree of sexual form dimorphism in *A. afarensis* remains significantly lower than in the *Gorilla* species and significantly higher than in *P. troglodytes* and *H. sapiens*, yet potentially does not differ from *P. paniscus* when including the predictions (Table S20). In shape space, the degree of sexual dimorphism in *A. afarensis* is still significantly higher than in *H. sapiens* and lower than in the *Gorilla* species when including predictions in the sample, but potentially also significantly lower than in the *Pan* species. The reduced shape differences between *A. afarensis* sexes are also illustrated in Figure 11b where male and female dental arcades are almost identical in shape when including predictions in the *A. afarensis* sample.

### 4.4 Sex assignments

Analyses were done on the assumption that published sex assignments for the *A. afarensis* specimens are correct, yet size and shape data are not always consistent. For example, the dental arcade of A.L. 277‐1 falls within the female range of size variation observed in this study, and it was previously noted that its mandibular corpus at M_1_ level is intermediate in height and its P_3_ is unicuspid, the typical configuration for *A. afarensis* females (Kimbel et al., 1985, 1994, 2004). Still, this specimen has been classified as male based on its overall large size and its wide C¯, P_3_, and M_2_ crowns (Leonard & Hegmon, 1987). The latter observations are less distinct for the alveolar data as the C¯ and M_2_ alveolar widths of A.L. 277‐1 fall at the lower end of the male range. Note that at least one small female, A.L. 315‐22, has a bicuspid P_3_ crown (Kimbel et al., 1994), demonstrating that this feature cannot always be linked with sex. Moreover, metaconid expression in the P_3_ of *A. afarensis* appears to be independent of size and is highly variable, ranging from absent to well‐developed and distinctly separated from the protoconid, with many specimens showing an intermediate condition (Delezene & Kimbel, 2011; Kimbel & Delezene, 2009).

The classification of A.L. 266‐1 as a female is based on its low mandibular corpus height and its narrow M_2_ crown (Kimbel et al., 2004; Leonard & Hegmon, 1987), although the latter is not obviously reflected in the alveolar width, which falls at the upper range of female variation. Moreover, this specimen has the largest dental arcade size of all females examined in this study. Canine metrics have not been taken into account before when assessing the sex of A.L. 266‐1, given that the crowns are missing. Even though the C¯ alveolar width of A.L. 266‐1 is intermediate in our sample, it does fall with the other female specimens. Nevertheless, this result should be considered with caution, given that the labial C¯ landmarks are estimated for this specimen. More convincing evidence for its female status is the P_3_ alveolar width of A.L. 266‐1, which is the second lowest in our entire sample.

Another specimen which deserves attention here is A.L. 400‐1a, which was sexed as potentially female on the basis of narrow C¯ crowns, noting its wide M_2_ crowns and intermediate P_3_ crown breadths and mandibular corpus height (Kimbel et al., 2004; Leonard & Hegmon, 1987). Leonard and Hegmon (1987) already pointed out that the canine is generally a better discriminator of sex in hominoids and thus A.L. 400‐1a is still considered a female.

Two specimens in our sample, A.L. 620‐1 and L.H. 4, have been assessed as male based on their large M_3_ crowns, yet their mandibular corpus heights at M_1_ level are intermediate and low, respectively (Kimbel et al., 2004). The overall dental arcade size and alveolar width of the C¯ and M_2_ consistently fall at the upper end of our sample for A.L. 620‐1, further validating its male status. In contrast, these measurements are intermediate for L.H. 4, and this specimen also has very narrow P_3_ alveoli, shedding some uncertainty on its sex assignment.

## 5 CONCLUSIONS

This study confirms that information from extant hominine species can be used to predict complementary dental arcades in fossils, allowing the maxillae of one hominin specimen to be compared with the mandible of another. Although morphological information about the rest of the maxilla is lost, the dental arcade can still provide clues about species affinities and enhance our understanding of *A. afarensis* as a species. Importantly, this study found that the degrees of size and shape variation are high in the dental arcade of *A. afarensis* in the context of extant hominine variation, with levels most similar to those observed in the *Gorilla* species. When increasing the sample size with evidence obtained from *A. afarensis* mandibles, the degree of shape variation potentially even surpasses that of *Gorilla*.

In practice, it is now possible to test whether the morphology of a fossil specimen falls within the range of variation observed for *A. afarensis*, assessing either the entire maxillary morphology or only its dental arcade when comparing a non‐associated mandible. When a specimen's morphology clearly exceeds the variation observed in *A. afarensis*, this provides a reasonable indication that it may not belong to this species. In contrast, when differences fall within the range of variation observed for *A. afarensis*, taxonomic interpretations become more complex, as overlapping morphological variation can occur between distinct species. In such cases, conspecificity cannot be assumed, but the finding may be consistent with it and should prompt further investigation. While the degree of variation observed for *A. afarensis* may not be representative of all fossil hominin taxa, it nonetheless serves as a valuable benchmark for assessing mid‐Pliocene specimens and provides a more contextually relevant reference for comparison than extant hominines, whose levels of variation have traditionally been used in taxonomic assessments.

## AUTHOR CONTRIBUTIONS

**Hester Hanegraef:** Methodology; data curation; investigation; formal analysis; visualization; resources; writing – original draft; writing – review and editing. **Romain David:** Methodology; software; formal analysis; writing – review and editing. **Fred Spoor:** Conceptualization; data curation; supervision; funding acquisition; project administration; resources; writing – review and editing.

## CONFLICT OF INTEREST STATEMENT

The authors declare no conflicts of interest.

## Supporting information


**Data S1.** Procrustes coordinates and natural logarithms of centroid sizes for the fossil and extant maxillae provided as a separate supplementary file in text format.

**Data S2.** Procrustes coordinates and natural logarithms of centroid sizes for the fossil and extant mandibles provided as a separate supplementary file in text format.

**Table S1.** Extant specimen list provided as a separate.

**Figure S1.** Supporting Information figures and tables.

## Data Availability

The landmark data used in this study are provided in the Supporting Information.
