# Supplementary material for: Integrating mandibular evidence to assess morphological variation of the Australopithecus afarensis maxilla
Source: Anat Rec (Hoboken). 2025 Jul 31;309(6):1483–505. doi: 10.1002/ar.70027 (PMC13138283; doi:10.1002/ar.70027)
Supplement: Supplementary file 4 — Figure S1. Supporting Information figures and tables. [file AR-309-1483-s001.docx]

**DATA S1.** Procrustes coordinates and natural logarithms of centroid sizes for the fossil and extant maxillae provided as a separate supplementary file in text format.

**DATA S2.** Procrustes coordinates and natural logarithms of centroid sizes for the fossil and extant mandibles provided as a separate supplementary file in text format.

**NOTE S1. Preservation and reconstruction of A.L. 128-23**

A.L. 128-23 is a partial right mandible of a late juvenile that retains the broken crowns of the I_2_–C̅, the intact crowns of the P_3_–M_2_, and the exposed crypt of the M_3_ (Figure S1). A complete description is published in White & Johanson (1982). An irregular anterior break truncates the corpus through the right I_2_ and then veers to the left, preserving a small inferior portion of the anterior corpus. Most of the ramus is lost. Several matrix-filled cracks result in a slight expansion of the entire corpus and a vertical crack runs at M_1_ level, but none require reconstruction.

To reconstruct the specimen (Figure S1), the right mandible was mirror-imaged across the midplane, which was determined based on the contour of the anterior basal corpus and the position of the genioglossal ridge, the inferior mental spines, and the digastric fossae, as well as the space necessary to accommodate the I_1_ and the orientation of the postcanine occlusal plane.

**NOTE S2. Preservation and reconstruction of A.L. 198-1**

A.L. 198-1 is a partial left mandible retaining the broken alveolus of the I_1_, the root of the I_2_, the intact crowns of the C̅–M_3_, and part of the M_4_ (distomolar) mesial roots (Figure S2). A complete description is published in White & Johanson (1982). The specimen is broken approximately along the midplane superiorly, although the anterior break veers to the left of the midplane inferiorly. The ramus is not preserved. A vertical crack at M_2_ level results in minimal distortion and does not require reconstruction.

To reconstruct the specimen (Figure S2), the left mandible was mirror-imaged across the midplane, which was determined based on the anterior break approximating the midplane superiorly, the contour of the anterior surface and postincisive plane, and the orientation of the postcanine occlusal plane.

**NOTE S3. Preservation and reconstruction of A.L. 266-1**

A.L. 266-1 is a partial mandible retaining the broken alveoli from the left C̅ to the right C̅ and the intact crowns of the left P_3_–M_1_ and the right P_3_–M_3_ (Figure S3). A complete description is published in White & Johanson (1982). A vertical break truncates the corpus directly distal of the left M_1_, while on the right the corpus is preserved up to the M_3_ and the root of the ramus is also present. A vertical crack at right C̅/P_3_ level results in minimal distortion and does not require reconstruction.

Given the good preservation of this specimen, no reconstruction was needed.

**NOTE S4. Preservation and reconstruction of A.L. 277-1**

A.L. 277-1 is a partial left mandible retaining the broken alveoli of the I_1_–I_2_ and the intact crowns of the C̅–M_2_ (Figure S4). A complete description is published in White & Johanson (1982). Only part of the corpus is preserved, with the anterior break approximating the midplane superiorly and the posterior break running through the M_3_ alveolus. A longitudinal crack results in minimal distortion and does not require reconstruction.

To reconstruct the specimen (Figure S4), the left mandible was mirror-imaged across the midplane, which was determined based on the anterior break that runs approximately along the midplane superiorly, the contour of the anterior basal corpus, the position of the genioglossal ridge and the inferior mental spines, and the orientation of the postcanine occlusal plane. The anterior surface and postincisive plane of the original and mirror-imaged mandible do not align perfectly as some of the bone has eroded and perfect symmetry is also not expected in real life.

**NOTE S5. Preservation and reconstruction of A.L. 288-1i**

A.L. 288-1i is an almost complete mandible recovered in several fragments that make up the corpus and rami (Figure S5). It is part of the skeleton known as ‘Lucy’ and a complete description is published in Johanson et al. (1982). The specimen retains the roots of the left I_1_–I_2_, the left P_4_–M_1_, and the right I_1_, the broken alveoli of the left C̅–P_3_ and the right I_2_–C̅, and the intact crowns of the right P_3_–M_3_. The left P_3_ and left M_3_ were found in isolation.

To reconstruct the dental arcade (Figure S6), four mandibular corpus fragments were segmented and repositioned slightly so that the fragments align better along their breaks (Figure S6a). The right posterior corpus fragment was then mirror-imaged and fitted with the incomplete left side of the mandible (Figure S6b).

**NOTE S6. Preservation and reconstruction of A.L. 315-22**

A.L. 315-22 is a partial right mandible retaining the broken alveoli of the I_1_–C̅ and the M_3_, the broken crowns of the P_3_ and M_1_, and the roots of the P_4_ and M_2_ (Figure S7). A complete description is published in Robinson (2003). The anterior break runs from the base of the corpus on the left side of the midplane and rises through the incisive septum. Only the root of the ramus is preserved. A crack at P_4_/M_1_ level results in minimal distortion and does not require reconstruction.

To reconstruct the specimen (Figure S7), the right mandible was mirror-imaged across the midplane, which was determined based on the contour of the anterior basal corpus and the position of the incisive septum, the supraspinous foramen, the fused mental spines, and the digastric fossae. However, no single midplane fits perfectly and therefore two reconstructions were created with one based on a good fit with the incisive septum and another based on a good fit with the contour of the anterior basal corpus. The impact of these reconstructions is examined in Note S21.

**NOTE S7. Preservation and reconstruction of A.L. 330-5**

A.L. 330-5 is a partial mandible retaining the broken alveoli of the left I_1_–I_2_ and the right I_1_–C̅, the broken crown of the left C̅, part of the left P_3_ mesial roots, the roots of the right P_3_, and the intact crowns of the right P_4_–M_3_ (Figure S8). A complete description is published in Robinson (2003). A vertical break truncates the corpus through the left P_3_, while on the right the corpus is preserved up to the M_3_ but very little of the ramus root remains. Several matrix-filled cracks result in a slight expansion of the entire corpus, but they result in minimal distortion and do not require reconstruction.

To reconstruct the specimen (Figure S9), a vertical crack at right I_1_ level was realigned and the entire mandible was then mirror-imaged across the midplane so that the anterior dental alveoli, anterior surface, postincisive plane, and base of the anterior corpus align on both sides and the postcanine occlusal plane is orientated correctly.

**NOTE S8. Preservation and reconstruction of A.L. 333w-60**

A.L. 333w-60 is a partial mandible retaining the broken crowns of the right I_1_–C̅, the broken alveoli of the left I_1_–C̅, and the intact crowns of the left P_3_–M_3_ (Figure S10). A complete description is published in White & Johanson (1982). A vertical break truncates the corpus through the right C̅, while on the left the corpus is preserved up to the M_3_ but the ramus is completely lost. The right anterior corpus is affected by plastic deformation. Several matrix-filled cracks result in a slight expansion of the entire corpus and a vertical crack runs at left I_2_/C̅ level, but they result in minimal distortion and do not require reconstruction.

To reconstruct the specimen (Figure S11), everything right of the midplane was removed and the remaining left mandible was mirror-imaged across that midplane, which was determined based on the position of the incisive septum, the supraspinous foramen, and the genioglossal ridge, as well as the orientation of the postcanine occlusal plane (Figure S11a). The right I_1_, I_2_, and C̅ crowns along with a small part of the alveolar process not affected by plastic deformation were segmented and fitted with the reconstructed mandible, and then mirror-imaged across the midplane (Figure S11b).

**NOTE S9. Preservation and reconstruction of A.L. 400-1a**

A.L. 400-1a comprises a partial left and right mandible with complete adult dentition, although only the root of the right I_1_ remains and the left P_3_ is broken (Figure S12). A complete description is published in White & Johanson (1982). The specimen was recovered in two parts that join perfectly along their break. The ramus is missing on both sides. Several cracks result in minimal distortion and do not require reconstruction.

To reconstruct the specimen (Figure S12), the left and right mandibular corpus fragments were joined along their break. Note that this specimen has a natural torsion of the dental arcade.

**NOTE S10. Preservation and reconstruction of A.L. 417-1ab**

The preservation and reconstruction procedure of the A.L. 417-1ab mandible is described in Hanegraef & Spoor (2025).

**NOTE S11. Preservation and reconstruction of A.L. 437-1**

A.L. 437-1 is a partial mandible retaining the roots of the right I_1_–I_2_ and the left I_1_–P_3_, the broken alveoli of the right C̅, and the broken crowns of the left P_4_–M_3_ (Figure S13). A complete description is published in Robinson (2003). A vertical break truncates the corpus through the right C̅ alveolus, while on the left the corpus is preserved up to the M_3_ yet nothing of the ramus remains. A vertical crack at P_4_ level results in minimal distortion and does not require reconstruction.

To reconstruct the specimen (Figure S13), the mandible was mirror-imaged across the midplane, which was determined based on the position of the incisive septum, the fused mental spines, and the digastric fossae. The original and mirror-imaged anterior dental alveoli, anterior surface, postincisive plane, and base of the anterior corpus align well.

**NOTE S12. Preservation and reconstruction of A.L. 437-2**

A.L. 437-2 is a partial mandible retaining the crowns from the left C̅ to the right M_3_, although most are broken and cracked (Figure S14). A complete description is published in Robinson (2003). A vertical break truncates the corpus distal of the left C̅, while on the right the corpus is present up to the M_3_ and the root of the ramus is preserved. Vertical cracks at left I_1_ level and right P_4_/M_1_ level result in minimal distortion and do not require reconstruction.

To reconstruct the specimen (Figure S14), the mandible was mirror-imaged across the midplane, which was determined based on the position of the incisive septum, the genioglossal ridge, and the fused inferior mental spines. The original and mirror-imaged anterior dentition, anterior surface, postincisive plane, and base of the anterior corpus align well.

**NOTE S13. Preservation and reconstruction of A.L. 438-1g**

A.L. 438-1g is a partial right mandible retaining the broken alveolus of the I_1_ and the roots of the I_2_–M_3_ (Figure S15). A complete description is published in Robinson (2003). An irregular anterior break truncates the corpus through the left I_1_ alveolus, which has disappeared due to in-vivo infill and bone resorption after the tooth was lost. Several fragments of the right ramus are preserved. A vertical crack at P_4_/M_1_ level results in minimal distortion and does not require reconstruction.

To reconstruct the dental arcade (Figure S16), the right mandibular corpus was segmented and mirror-imaged across the midplane, which was determined based on the position of the incisive septum, the supraspinous foramen, and the fused mental spines. However, the original right mandible and mirror-imaged left mandible can be aligned in more than one way and therefore two plausible reconstructions were created, varying in dental arcade width. The impact of these reconstructions is examined in Note S21.

**NOTE S14. Preservation and reconstruction of A.L. 444-2b**

The preservation and segmentation of the A.L. 444-2b mandible is described in Hanegraef & Spoor (2025). A midplane based on a good fit with the postincisive plane of the mandible resulted in the most plausible reconstruction of the maxillae, indicating that plastic deformation probably affects the left anterior part of the mandible. To reconstruct the mandibular dental arcade (Figure S17), everything left of the midplane was removed from the mandible and the remaining right side was mirror-imaged across the midplane (Figure S17a, e). The reconstructed maxilla was again fitted with the reconstructed mandible (Figure S17b, f), after which the segmented posterior part of the mandibular corpus that holds the right M_2_ and M_3_ was fitted with the reconstructed mandible based on good occlusion with the maxillary teeth and then mirror-imaged across the midplane (Figure S17c, g). The segmented fragments of the left I_1_ and right C̅ were then fitted to the reconstructed mandible and mirror-imaged (Figure S17d, h).

**NOTE S15. Preservation and reconstruction of A.L. 620-1**

A.L. 620-1 is a partial left mandible retaining the roots of the I_1_–I_2_, the broken alveolus of the C̅, the broken crowns of the P_3_–M_2_, and the intact crown of the M_3_ (Figure S18). A complete description is published in Robinson (2003). An anterior break runs from the base of the corpus on the right side of the midplane and rises through the left I_1_ alveolus. The ramus is not preserved.

To reconstruct the specimen (Figure S18), the left mandible was mirror-imaged across the midplane, which was determined based on the position of the genioglossal ridge, the fused inferior mental spines, and the digastric fossae, as well as the orientation of the postcanine occlusal plane and the contour of the anterior surface, postincisive plane, and base of the anterior corpus.

**NOTE S16. Preservation and reconstruction of A.L. 822-1**

The preservation and reconstruction procedure of the A.L. 822-1 mandible is described in Hanegraef & Spoor (2025).

**NOTE S17. Preservation and reconstruction of A.L. 1045-1**

A.L. 1045-1 is an undescribed partial mandible retaining the broken alveoli of the left I_1_–P_3_, the roots of the right I_1_–C̅ and the left M_3_, the broken crowns of the left P_4_–M_2_ and the right P_4_–M_1_, and the intact crowns of the right P_3_ and the right M_2_–M_3_ (Figure S19). The root of the ramus is preserved on both sides. The left corpus is affected by taphonomic deformation, with the dental arcade angle distorted as a result of bilateral compression.

To reconstruct the specimen (Figure S20), everything left of the midplane was removed and the remaining right mandible was mirror-imaged across that midplane, which was determined based on the position of the incisive septum, the supraspinous foramen, and the fused mental spines, as well as the orientation of the postcanine occlusal plane and the contour of the anterior surface, postincisive plane, and base of the anterior corpus.

**NOTE S18. Preservation and reconstruction of A.L. 1496-1**

A.L. 1496-1 is an undescribed partial mandible retaining the broken alveoli of the right I_1_–C̅, the roots of the left I_1_–P_4_, and the variably preserved crowns of the left M_1_–M_3_ and the right P_3_–M_3_ (Figure S21). Only the roots of the rami are preserved. Vertical cracks at right C̅ level, left C̅ level, and left M_1_/M_2_ level result in minimal distortion and do not require reconstruction.

Given the good preservation of this specimen, no reconstruction was needed.

**NOTE S19. Preservation and reconstruction of A.L. 1901-1**

A.L. 1901-1 is an undescribed partial mandible consisting of four major fragments: (1) the anterior basal corpus; (2) the right corpus retaining the roots of the I_2_–P_3_, the broken crowns of the P_4_–M_3_, and an underdeveloped M_4_ (distomolar); (3) the anterior left corpus retaining the roots of the I_1_–I_2_, the broken alveolus of the P_3_, and the broken crowns of the C̅ and the P_4_–M_1_; and (4) the posterior left corpus retaining the broken crown of the M_3_ and a fully developed M_4_ (Figure S22). The ramus is not preserved on either side. Matrix-filled cracks result in the expansion of the entire corpus and in gaps between the teeth and alveolar process. These cracks cannot be reconstructed and should be kept in mind during the analyses.

To reconstruct the specimen (Figure S22), the right and anterior left mandibular corpus fragments were aligned with the anterior basal corpus based on joints that fit perfectly together (Figure S22a, e). The right corpus fragment was then mirror-imaged and aligned with the anterior left corpus fragment to be able to position the posterior left corpus fragment based on the overlap between the M_3_ and alveolar process (Figure S22b, f). The mirror-imaged left mandibular corpus fragment was then removed from the reconstruction (Figure S22c, g). Lastly, the labially displaced right C̅ was segmented and moved in the correct position in its alveolus (Figure S22d, h).

**NOTE S20. Preservation and reconstruction of L.H. 4**

L.H. 4 is a partial mandible retaining the broken alveoli of the left I_1_–C̅ and the right I_1_, the roots of the left P_3_, the broken crowns of the right C̅, and the intact crowns of the left P_4_–M_2_ and the right P_3_–M_3_ (Figure S23). A complete description is published in White (1977). The corpus was originally recovered in two parts that were subsequently joined along their break, although perfect alignment was not possible due to the slight warping of the bone near the centre of the broken right symphyseal surface. Both rami are missing. The right I_2_ was lost during life and resorptive alveolar pathology has obliterated its alveolus.

To reconstruct the dental arcade (Figure S24), the labially displaced right C̅ was segmented and moved in the correct position in its alveolus.

**NOTE S21. Impact of multiple and less reliable reconstructions**

Principal component analyses of the *A. afarensis* maxillary and mandibular dental arcades show that the plausible reconstructions of A.L. 442-1, A.L. 315-22, and A.L. 438-1g plot close together in shape space, although less so for the latter specimen (Figure S25). Nevertheless, the minimum Procrustes shape distance between *A. afarensis* specimens is substantially larger than the Procrustes shape distances between plausible reconstructions (Table S2). Given these small shape differences, average landmark configurations of the plausible reconstructions were used in the analyses.

Moreover, the dental arcade reconstructions of A.L. 128-23 and A.L. 198-1 are less reliable than those of the other mandibles given that so little of their midplane characteristics are preserved. Still, their reconstructions are constrained by morphology and both plot within the shape space of the other *A. afarensis* specimens (Figure S25b).

**NOTE S22. Accuracy of landmark estimations**

For four *A. afarensis* mandibles (A.L. 198-1, A.L. 315-22, A.L. 438-1g, and A.L. 1045-1), reasonable inferences could be made about missing landmarks based on the preserved morphology (Table 1). These morphological estimations were then checked against estimations based on thin-plate spline interpolation (Figure S26). Landmark locations only differ slightly between the two methods, except for A.L. 315-22 where thin-plate spline interpolation results in unrealistic landmark estimations (Figure S26c–d). Note that the labial alveolar margins of the incisors and canines were damaged postmortem in this specimen as is evident from the rounded edges. There is thus a degree of uncertainty about the inferosuperior location of the labial landmarks in A.L. 315-22, but slight deviations from the original morphology are not expected to have a substantial effect on the results as this would not alter the overall architecture of the dental arcade shape. Given that landmark estimations based on the preserved morphology do not rely on shape information from other specimens and estimations based on thin-plate spline interpolation sometimes result in unrealistic landmark locations, the former were used in the analyses.

For eight *A. afarensis* mandibles (A.L. 128-23, A.L. 266-1, A.L. 277-1, A.L. 288-1i, A.L. 330-5, A.L. 620-1, A.L. 1496-1, and L.H. 4), missing landmarks were estimated using thin-plate spline interpolation (Table 1). Locations of estimated landmarks are considered sufficiently accurate in most cases (Figure S27a–e), including for the L.H. 4 mandible (Figure S27h) which is geologically older and from a different geographical area as the Hadar mandibles used to obtain the reference specimen. On the other hand, the estimated location of labial landmarks for A.L. 620-1 (Figure S27f) and A.L. 1496-1 (Figure S27g) are not plausible. These landmarks were manually adjusted in the transverse plane to a location more consistent with morphological clues, including the likely trajectory of the alveolar process (Figure S28). When assessing the dental arcade shape of all available *A. afarensis* mandibles, A.L. 620-1 and A.L. 1496-1 plot at opposite extremes on PC1 (Figure S25b), representing a relatively more U-shaped dental arcade on the negative end and a relatively more V-shaped arcade on the positive end, respectively. These shapes are unlikely to be affected by the location of the anterior labial landmarks and therefore it is not expected that the manual landmark adjustments will impact results substantially.

**NOTE S23. R code for extant and fossil predictions**

```
#This script assumes the following objects are preloaded:
# - csize.maxillae.extant.original: named numeric vector of extant maxillary dental arcade
#                                   centroid sizes
# - csize.mandibles.extant.original: named numeric vector of extant mandibular dental
#                                    arcade centroid sizes
# - csize.mandibles.fossil.original: named numeric vector of A. afarensis mandibular dental
#                                    arcade centroid sizes
# - gpa.coords.maxillae.extant.original: named 3D array of extant maxillary dental arcade
#                                        shapes
# - gpa.coords.mandibles.extant.original: named 3D array of extant mandibular dental arcade
#                                         shapes
# - gpa.coords.mandibles.fossil.original: named 3D array of A. afarensis mandibular dental
#                                         arcade shapes

library(rgl)
library(RRPP)
library(Matrix)
library(geomorph)
library(MASS)

####SIZE PREDICTIONS####

#create an empty vector to store predicted extant maxillary dental arcade centroid sizes
csize.maxillae.extant.prediction<-rep(NA, length(csize.maxillae.extant.original))
names(csize.maxillae.extant.prediction)<-names(csize.maxillae.extant.original)

#construct linear regression with leave-one-out cross validation using the extant sample and predict maxillary dental arcade centroid sizes of extant specimens from their mandibular dental arcade centroid sizes
for (i in 1:length(csize.maxillae.extant.original)) {
  Y<-csize.maxillae.extant.original[-i]
  X<-csize.mandibles.extant.original[-i]
  fit.size<-lm(Y~X)

  newdata<-data.frame(X=csize.mandibles.extant.original[i])
  csize.maxillae.extant.prediction[i]<-predict(fit.size, newdata=newdata)
}

#construct linear regression without leave-one-out cross validation using the extant sample
Y<-csize.maxillae.extant.original
X<-csize.mandibles.extant.original
fit.size<-lm(Y~X)

#predict maxillary dental arcade centroid sizes of A. afarensis specimens from their mandibular dental arcade centroid sizes using the linear regression without leave-one-out cross validation
csize.maxillae.fossil.prediction<-
    predict(fit.size, newdata=data.frame(X=csize.mandibles.fossil.original))

####SHAPE PREDICTIONS####

#obtain principal component scores for the original extant mandibular dental arcade shapes
PCA.mandibles.extant.original<-prcomp(two.d.array(gpa.coords.mandibles.extant.original))
pc.scores.mandibles.extant.original<-PCA.mandibles.extant.original$x

#create an empty matrix to store predicted extant maxillary dental arcade shapes
gpa.coords.maxillae.extant.prediction<-
   matrix(NA, nrow=dim(gpa.coords.maxillae.extant.original)[3], ncol=56*3)
rownames(gpa.coords.maxillae.extant.prediction)<-
   rownames(two.d.array(gpa.coords.maxillae.extant.original))
colnames(gpa.coords.maxillae.extant.prediction)<-
   colnames(two.d.array(gpa.coords.maxillae.extant.original))

#construct multiple multivariate regression with leave-one-out cross validation using the extant sample and predict maxillary dental arcade shapes of extant specimens from their mandibular dental arcade shapes
for (i in 1:dim(gpa.coords.maxillae.extant.original)[3]) {
  Y<-two.d.array(gpa.coords.maxillae.extant.original)[-i,]
  X1<-pc.scores.mandibles.extant.original[-i,1]
  X2<-pc.scores.mandibles.extant.original[-i,2]
  X3<-pc.scores.mandibles.extant.original[-i,3]
  X4<-pc.scores.mandibles.extant.original[-i,4]
  X5<-pc.scores.mandibles.extant.original[-i,5]
  X6<-pc.scores.mandibles.extant.original[-i,6]
  X7<-pc.scores.mandibles.extant.original[-i,7]
  X8<-pc.scores.mandibles.extant.original[-i,8]
  X9<-pc.scores.mandibles.extant.original[-i,9]
  X10<-pc.scores.mandibles.extant.original[-i,10]
  fit.shape<-lm(Y~X1+X2+X3+X4+X5+X6+X7+X8+X9+X10)

  newdata<-data.frame(X1=pc.scores.mandibles.extant.original[i,1],
                      X2=pc.scores.mandibles.extant.original[i,2],
                      X3=pc.scores.mandibles.extant.original[i,3],
                      X4=pc.scores.mandibles.extant.original[i,4],
                      X5=pc.scores.mandibles.extant.original[i,5],
                      X6=pc.scores.mandibles.extant.original[i,6],
                      X7=pc.scores.mandibles.extant.original[i,7],
                      X8=pc.scores.mandibles.extant.original[i,8],
                      X9=pc.scores.mandibles.extant.original[i,9],
                      X10=pc.scores.mandibles.extant.original[i,10])
  gpa.coords.maxillae.extant.prediction[i,]<-predict(fit.shape, newdata=newdata)
}

#construct multiple multivariate regression without leave-one-out cross validation using the extant sample
Y<-two.d.array(gpa.coords.maxillae.extant.original)
X1<-pc.scores.mandibles.extant.original[,1]
X2<-pc.scores.mandibles.extant.original[,2]
X3<-pc.scores.mandibles.extant.original[,3]
X4<-pc.scores.mandibles.extant.original[,4]
X5<-pc.scores.mandibles.extant.original[,5]
X6<-pc.scores.mandibles.extant.original[,6]
X7<-pc.scores.mandibles.extant.original[,7]
X8<-pc.scores.mandibles.extant.original[,8]
X9<-pc.scores.mandibles.extant.original[,9]
X10<-pc.scores.mandibles.extant.original[,10]
fit.shape<-lm(Y~X1+X2+X3+X4+X5+X6+X7+X8+X9+X10)

#project original A. afarensis mandibular dental arcades into shape space of the original extant mandibular dental arcades
pc.scores.mandibles.fossil.original<-predict(PCA.mandibles.extant.original,
    two.d.array(gpa.coords.mandibles.fossil.original))

#create an empty matrix to store predicted A. afarensis maxillary dental arcade shapes
gpa.coords.maxillae.fossil.prediction<-
    matrix(NA, nrow=dim(pc.scores.mandibles.fossil.original)[1], ncol=56*3)
rownames(gpa.coords.maxillae.fossil.prediction)<-
    rownames(pc.scores.mandibles.fossil.original)
colnames(gpa.coords.maxillae.fossil.prediction)<-
    colnames(two.d.array(gpa.coords.mandibles.fossil.original))

#predict maxillary dental arcade shapes of A. afarensis specimens from their mandibular dental arcade shapes using the multiple multivariate regression without leave-one-out cross validation
for (i in 1:dim(pc.scores.mandibles.fossil.original)[1]) {
  newdata<-data.frame(X1=pc.scores.mandibles.fossil.original[i,1],
                      X2=pc.scores.mandibles.fossil.original[i,2],
                      X3=pc.scores.mandibles.fossil.original[i,3],
                      X4=pc.scores.mandibles.fossil.original[i,4],
                      X5=pc.scores.mandibles.fossil.original[i,5],
                      X6=pc.scores.mandibles.fossil.original[i,6],
                      X7=pc.scores.mandibles.fossil.original[i,7],
                      X8=pc.scores.mandibles.fossil.original[i,8],
                      X9=pc.scores.mandibles.fossil.original[i,9],
                      X10=pc.scores.mandibles.fossil.original[i,10])
  gpa.coords.maxillae.fossil.prediction[i,]<-predict(fit.shape, newdata=newdata)
}

####PREDICTED SHAPE CORRECTIONS####
#this step estimates uncertainty around predicted fossil shape by modeling prediction error as a multivariate normal distribution derived from extant residuals

#obtain principal component scores for the original extant maxillary dental arcade shapes
PCA.maxillae.extant.original<-prcomp(two.d.array(gpa.coords.maxillae.extant.original))
pc.scores.maxillae.extant.original<-PCA.maxillae.extant.original$x

#project predicted extant maxillary dental arcades into shape space of the original extant maxillary dental arcades
pc.scores.maxillae.extant.prediction<-
    predict(PCA.maxillae.extant.original, gpa.coords.maxillae.extant.prediction)

#calculate residual errors
residual.errors<-pc.scores.maxillae.extant.original-pc.scores.maxillae.extant.prediction

#create multivariate normal distribution from residual errors and extract 1000 random samples
set.seed(100)
residual.errors.resampled<-
    mvrnorm(n=1000, mu=colMeans(residual.errors), cov(residual.errors))

#project predicted A. afarensis maxillary dental arcades into shape space of the original extant maxillary dental arcades
pc.scores.maxillae.fossil.prediction<-
    predict(PCA.maxillae.extant.original, gpa.coords.maxillae.fossil.prediction)

#select A.L. 288-1i as an example
pc.scores.maxillae.AL288.1i.prediction<-
    pc.scores.maxillae.fossil.prediction["A.L. 288-1i",]

#create an empty matrix to store corrected projected principal component scores of the A.L. 288-1i predicted maxillary dental arcade shape
pc.scores.maxillae.AL288.1i.prediction.corrected<-
     matrix(NA, nrow=1000, ncol=length(pc.scores.maxillae.AL288.1i.prediction))

#add 1000 randomly sampled residual errors to the projected principal component scores of the A.L. 288-1i predicted maxillary dental arcade shape
for (i in 1:1000) {
  pc.scores.maxillae.AL288.1i.prediction.corrected[i,]<-
      pc.scores.maxillae.AL288.1i.prediction+residual.errors.resampled[i,]
}

#create an empty array to store corrected Procrustes coordinates of the A.L. 288-1i predicted maxillary dental arcade shape
gpa.coords.maxillae.AL288.1i.prediction.corrected<-array(NA, dim=c(56,3,1000))
dimnames(gpa.coords.maxillae.AL288.1i.prediction.corrected)[[1]]<-
    dimnames(gpa.coords.maxillae.extant.original)[[1]]
dimnames(gpa.coords.maxillae.AL288.1i.prediction.corrected)[[2]]<-
    dimnames(gpa.coords.maxillae.extant.original)[[2]]
dimnames(gpa.coords.maxillae.AL288.1i.prediction.corrected)[[3]]<-
    paste("A.L. 288-1i_corrected_prediction_", c(1:1000), sep="")

#predict Procrustes coordinates from the 1000 corrected projected principal component scores of the A.L. 288-1i predicted maxillary dental arcade shape
for (i in 1:1000) {
  predicted.shape<-shape.predictor(gpa.coords.maxillae.extant.original,
      x=pc.scores.maxillae.extant.original, Intercept=TRUE,
      value=pc.scores.maxillae.AL288.1i.prediction.corrected[i,])
  gpa.coords.maxillae.AL288.1i.prediction.corrected[,,i]<-predicted.shape$value
}

#calculate mean of the 1000 corrected Procrustes coordinates of the A.L. 288-1i predicted maxillary dental arcade shape
gpa.coords.maxillae.AL288.1i.prediction.corrected.mean<-
    mshape(gpa.coords.maxillae.AL288.1i.prediction.corrected)
```

**FIGURE S1** The reconstructed A.L. 128-23 mandible in occlusal (a), inferior (b), frontal (c), posterior (d), lateral (e), and medial (f) view. Scale bar 1 cm.

**FIGURE S2** The reconstructed A.L. 198-1 mandible in occlusal (a), inferior (b), frontal (c), posterior (d), lateral (e), and medial (f) view. Scale bar 1 cm.

**FIGURE S3** The A.L. 266-1 mandible in occlusal (a), inferior (b), frontal (c), posterior (d), left lateral (e), and right lateral (f) view. Scale bar 1 cm.

**FIGURE S4** The reconstructed A.L. 277-1 mandible in occlusal (a), inferior (b), frontal (c), posterior (d), lateral (e), and medial (f) view. Scale bar 1 cm.

**FIGURE S5** The A.L. 288-1i mandible in occlusal (a), inferior (b), frontal (c), posterior (d), left lateral (e), and right lateral (f) view. Scale bar 1 cm.

**FIGURE S6** The A.L. 288-1i reconstruction in occlusal view, showing the realignment of four mandibular corpus fragments (a) and the mirror-imaging of the right posterior corpus fragment (b). Scale bar 1 cm.

**FIGURE S7** The reconstructed A.L. 315-22 mandible in occlusal (a–c), inferior (d–f), frontal (g–i), posterior (j–l), lateral (m), and medial (n) view. Two plausible spatial alignments of the mirror-imaged right mandible are shown in blue and yellow, highlighting the differences in the superimposed views (c, f, i, l). Scale bar 1 cm.

**FIGURE S8** The A.L. 330-5 mandible in occlusal (a), inferior (b), frontal (c), posterior (d), lateral (e), and medial (f) view. Scale bar 1 cm.

**FIGURE S9** The A.L. 330-5 reconstruction in occlusal view, showing the original mandible with a vertical crack (red arrow) at right I_1_ level (a), the realignment of this crack (b), and the mirror-imaging of the mandibular corpus (c). Scale bar 1 cm.

**FIGURE S10** The A.L. 333w-60 mandible in occlusal (a), inferior (b), frontal (c), posterior (d), lateral (e), and medial (f) view. Scale bar 1 cm.

**FIGURE S11** The A.L. 333w-60 reconstruction in occlusal view (a) and the addition of the anterior dental fragments (b). Scale bar 1 cm.

**FIGURE S12** The reconstructed A.L. 400-1a mandible in occlusal (a), inferior (b), frontal (c), posterior (d), left lateral (e), right lateral (f), left medial (g), and right medial (h) view. Scale bar 1 cm.

**FIGURE S13** The reconstructed A.L. 437-1 mandible in occlusal (a), inferior (b), frontal (c), posterior (d), lateral (e), and medial (f) view. Scale bar 1 cm.

**FIGURE S14** The reconstructed A.L. 437-2 mandible in occlusal (a), inferior (b), frontal (c), posterior (d), lateral (e), and medial (f) view. Scale bar 1 cm.

**FIGURE S15** The A.L. 438-1g mandible in occlusal (a), inferior (b), frontal (c), posterior (d), lateral (e), and medial (f) view. Scale bar 1 cm.

**FIGURE S16** The reconstructed A.L. 438-1g mandible in occlusal (a–c), inferior (d–f), frontal (g–i), posterior (j–l), lateral (m), and medial (n) view. Two plausible spatial alignments of the mirror-imaged right mandible are shown in blue and yellow, highlighting the differences in the superimposed views (c, f, i, l). Scale bar 1 cm.

**FIGURE S17** The A.L. 444-2b reconstruction in frontal (a–d) and lateral (e–h) view. Scale bar 1 cm.

**FIGURE S18** The reconstructed A.L. 620-1 mandible in occlusal (a), inferior (b), frontal (c), posterior (d), lateral (e), and medial (f) view. Scale bar 1 cm.

**FIGURE S19** The A.L. 1045-1 mandible in occlusal (a), inferior (b), frontal (c), posterior (d), left lateral (e), and right lateral (f) view. Scale bar 1 cm.

**FIGURE S20** The original (a) and reconstructed (b) A.L. 1045-1 mandible in occlusal view. Scale bar 1 cm.

**FIGURE S21** The A.L. 1496-1 mandible in occlusal (a), inferior (b), frontal (c), posterior (d), left lateral (e), and right lateral (f) view. Scale bar 1 cm.

**FIGURE S22** The A.L. 1901-1 reconstruction in occlusal (a–d) and frontal (e–h) view. Scale bar 1 cm.

**FIGURE S23** The L.H. 4 mandible in occlusal (a), inferior (b), frontal (c), posterior (d), left lateral (e), and right lateral (f) view. Scale bar 1 cm.

**FIGURE S24** L.H. 4 reconstruction in occlusal view, showing the original mandible with a labially displaced right C̅ (a) and the repositioning of this tooth in its alveolar socket (b). Scale bar 1 cm.

**FIGURE S25** Plots of PC1 and PC2 showing maxillary (a) and mandibular (b) dental arcade shapes of *A. afarensis*. Three plausible reconstructions are shown for the A.L. 442-1 maxillae and two plausible reconstructions for the A.L. 315-22 and A.L. 438-1g mandibles. Wireframes show shape differences along PC1 and PC2 in occlusal view.

**FIGURE S26** The reconstructed A.L. 198-1 (a–b), A.L. 315-22 (c–d), A.L. 438-1g (e–f), and 1045-1 (g–h) mandibles in occlusal (top) and frontal (bottom) view. Original landmarks are shown as golden spheres and estimated landmarks using thin-plate spline interpolation as red spheres. Scale bar 1 cm.

**FIGURE S27** The reconstructed A.L. 128-23 (a), A.L. 266-1 (b), A.L.277-1 (c), A.L. 288-1i (d), A.L. 330-5 (e), A.L. 620-1 (f), A.L. 1496-1 (g), and L.H. 4 (h) mandibles in occlusal view. Original landmarks are shown as golden spheres, estimated landmarks using bilateral symmetry as blue spheres, estimated landmarks using thin-plate spline interpolation as red spheres, and manually adjusted landmarks as green spheres. Scale bar 1 cm.

**FIGURE S28** Mesial view of labiolingual µCT slices through the left I_1_ (a) and left C̅ (b) of the A.L. 620-1 mandible and the left I_1_ (c), left I_2_ (d), left C̅ (e), right I_1_ (f), right I_2_ (g), and right C̅ (h) of the A.L. 1496-1 mandible. Original landmarks are shown as golden spheres, estimated landmarks using bilateral symmetry as blue spheres, estimated landmarks using thin-plate spline interpolation as red spheres, and manually adjusted landmarks as green spheres. Scale bar 1 cm.

**FIGURE S29** Covariation between the centroid sizes of the maxillary and mandibular dental arcades of *H. sapiens* (red sideways triangles), *P. troglodytes* (blue diamonds), *P. paniscus* (yellow upwards triangles), *G. gorilla* (green squares), and *G. beringei* (pink downwards triangles). All species scatter along a diagonal.

**FIGURE S30** Two-block partial least squares analysis between the maxillary and mandibular dental arcade shapes of *H. sapiens* (red sideways triangles), *P. troglodytes* (blue diamonds), *P. paniscus* (yellow upwards triangles), *G. gorilla* (green squares), and *G. beringei* (pink downwards triangles). Wireframes show shape differences along PLS1 (a) and PLS2 (b) in occlusal view. All species scatter along the diagonal.

**FIGURE S31** Comparison between original and predicted natural logarithms of centroid sizes for the maxillary dental arcades of *A. afarensis* (grey), *H. sapiens* (red), *P. troglodytes* (blue), *P. paniscus* (yellow), *G. gorilla* (green), and *G. beringei* (pink).

**FIGURE S32** Plot of PC1 and PC2 showing maxillary dental arcade shapes of *H. sapiens* (red sideways triangles), *P. troglodytes* (blue diamonds), *P. paniscus* (yellow upwards triangles), *G. gorilla* (green squares), and *G. beringei* (pink downwards triangles). Arrows point from the original to the predicted maxillary dental arcade shapes.

**FIGURE S33** Prediction error for *H. sapiens* (red), *P. troglodytes* (blue), *P. paniscus* (yellow), *G. gorilla* (green), and *G. beringei* (pink). Wireframes on the top show the smallest prediction errors (individuals with the smallest Procrustes shape distance between their original and predicted maxillary dental arcades), and wireframes on the bottom show the largest prediction errors (individuals with the largest Procrustes shape distance between their original and predicted maxillary dental arcades). Original maxillary dental arcade shapes are shown in black and predictions in colour.

**FIGURE S34** Prediction error for three *A. afarensis* specimens with associated upper and lower jaws. Original maxillary dental arcade shapes are shown in black and mean corrected predictions in grey.

**FIGURE S35** Temporal trends in the PC1 and PC2 scores, based on the placement of *A. afarensis* specimens in the stratigraphic units of Laetoli and Hadar. Original maxillary dental arcades are shown as filled black circles and mean corrected predictions as open black circles. See Table 1 for *A. afarensis* specimen numbers.

**FIGURE S36** Temporal trends in the natural logarithms of centroid sizes, based on the placement of *A. afarensis* specimens in the stratigraphic units of Laetoli and Hadar. Males are shown as black squares, females as grey circles, and specimens with unknown sex as open black diamonds.

**FIGURE S37** Natural logarithms of centroid sizes of *A. afarensis* mandibles and maxillae, ordered from smallest to largest value. Males are shown as black squares, females as grey circles, and specimens with unknown sex as open black diamonds.

**FIGURE S38** Absolute labiolingual widths of the canine alveoli of *A. afarensis* mandibles and maxillae, ordered from smallest to largest value. Males are shown as black squares, females as grey circles, and specimens with unknown sex as open black diamonds. Specimens with an asterisk are those for which the labial landmark of the canine alveolus was estimated.

**FIGURE S39** Absolute buccolingual widths of the third premolar alveoli of *A. afarensis* mandibles and maxillae, ordered from smallest to largest value. Males are shown as black squares, females as grey circles, and specimens with unknown sex as open black diamonds. Specimens with an asterisk are those for which the buccal landmark of the third premolar alveolus was estimated.

**FIGURE S40** Absolute buccolingual widths of the second molar alveoli of *A. afarensis* mandibles and maxillae, ordered from smallest to largest value. Males are shown as black squares, females as grey circles, and specimens with unknown sex as open black diamonds. Specimens with an asterisk are those for which the lingual landmark of the second molar alveolus was estimated.

**FIGURE S41** Sexual dimorphism trends between the maxillary dental arcades of males (black squares) and females (coloured circles) on PC1 and PC2 in form (a–e) and shape (f–j) space for each extant hominine species. Colours as in Figure 3.

**FIGURE S42** Differences between the dental arcade shapes of the *A. afarensis* late juveniles A.L. 486-1 (a–b) and A.L. 128-23 (c–d) and the mean maxillary and mandibular adult shape (black), respectively. Wireframes are shown in occlusal (top) and lateral (bottom) view. Shapes are rotated to minimise the distance between postcanine alveolar landmarks.

**FIGURE S43** Temporal trends in mandibular corpus heights at M_1_ (a) and M_2_ (b) for *A. afarensis* specimens from various localities. Specimens are plotted at the midpoint of their geological age range (see Table S20).

**TABLE S1** Extant specimen list provided as a separate supplementary file in Excel format.

**TABLE S2** Minimum Procrustes shape distances between *A. afarensis* maxillae and mandibles compared with the Procrustes shape distances between plausible reconstructions (Rec).

|  | **Procrustes shape distance** |
| --- | --- |
| **Maxilla** |  |
| Minimum between *A. afarensis* specimens | 0.05126 |
| A.L. 442-1 reconstructions (Rec1–Rec2) | 0.01440 |
| A.L. 442-1 reconstructions (Rec1–Rec3) | 0.01789 |
| A.L. 442-1 reconstructions (Rec2–Rec3) | 0.00557 |
| **Mandible** |  |
| Minimum between *A. afarensis* specimens | 0.04887 |
| A.L. 315-22 reconstructions (Rec1–Rec2) | 0.02306 |
| A.L. 438-1g reconstructions (Rec1–Rec2) | 0.02871 |

**TABLE S3** List of bilateral landmarks used to capture the dental arcade shape.

| **No.** | **Landmark definition** |
| --- | --- |
| 1–4 | I^1^/I_1_, left: mesial, distal, labial, and lingual sides of the alveolar margin. |
| 5–8 | I^1^/I_1_, right: mesial, distal, labial, and lingual sides of the alveolar margin. |
| 9–12 | I^2^/I_2_, left: mesial, distal, labial, and lingual sides of the alveolar margin. |
| 13–16 | I^2^/I_2_, right: mesial, distal, labial, and lingual sides of the alveolar margin. |
| 17–20 | C̲/C̅, left: mesial, distal, labial, and lingual sides of the alveolar margin. |
| 21–24 | C̲/C̅, right: mesial, distal, labial, and lingual sides of the alveolar margin. |
| 25–28 | P^3^/P_3_, left: mesial, distal, buccal, and lingual sides of the alveolar margin. ^a^ |
| 29–32 | P^3^/P_3_, right: mesial, distal, buccal, and lingual sides of the alveolar margin. ^a^ |
| 33–36 | P^4^/P_4_, left: mesial, distal, buccal, and lingual sides of the alveolar margin. ^a^ |
| 37–40 | P^4^/P_4_, right: mesial, distal, buccal, and lingual sides of the alveolar margin. ^a^ |
| 41–44 | M^1^/M_1_, left: mesial, distal, buccal, and lingual sides of the alveolar margin. ^a^ |
| 45–48 | M^1^/M_1_, right: mesial, distal, buccal, and lingual sides of the alveolar margin. ^a^ |
| 49–52 | M^2^/M_2_, left: mesial, distal, buccal, and lingual sides of the alveolar margin. ^a^ |
| 53–56 | M^2^/M_2_, right: mesial, distal, buccal, and lingual sides of the alveolar margin. ^a^ |

a When two buccal and/or two lingual roots were present, two landmarks were placed buccally and/or two lingually at the most exposed position of each root and their average was used in the analyses.

**TABLE S4** Results of the principal component analysis comparing mandibular dental arcade shape differences between extant hominine species.

|  | **Eigenvalue** | **% Total variance** | **Cumulative %** |
| --- | --- | --- | --- |
| PC1 | 0.01555 | 77.29 | 77.29 |
| PC2 | 0.00241 | 11.97 | 89.26 |
| PC3 | 0.00037 | 01.82 | 91.09 |
| PC4 | 0.00025 | 01.25 | 92.34 |
| PC5 | 0.00018 | 00.91 | 93.24 |
| PC6 | 0.00014 | 00.71 | 93.95 |
| PC7 | 0.00011 | 00.55 | 94.50 |
| PC8 | 0.00009 | 00.46 | 94.95 |
| PC9 | 0.00008 | 00.42 | 95.37 |
| PC10 | 0.00008 | 00.37 | 95.74 |

**TABLE S5** R packages and functions.

|  | Description |
| --- | --- |
| geomorph (Adams et al., 2025; Baken et al., 2021; Collyer & Adams, 2018, 2025) | |
| estimate.missing | Estimates the location of missing landmarks using thin-plate spline interpolation (method = “TPS”). |
| gpagen | Performs a generalised Procrustes analysis. |
| interlmkdist | Calculates linear distances between landmark coordinates. |
| mshape | Estimates the mean shape for a set of Procrustes aligned specimens. |
| plotRefToTarget | Plots shape differences between a reference and target specimen based on two matrices of landmark coordinates. |
| procD.lm | Performs a multivariate regression with permutation procedures. |
| shape.predictor | Estimates shape variables based on principal component scores (Intercept = TRUE). |
| MAd (Del Re & Hoyt, 2025) | |
| F_to_d | Converts F-statistics to Cohen’s d. |
| MASS (Ripley & Venables, 2025; Venables & Ripley, 2002) | |
| mvrnorm | Simulates samples from a multivariate normal distribution. |
| Morpho (Schlager, 2017, 2025) | |
| crossProduct | Calculates the orthogonal complement of a 3D vector. |
| fixLMmirror | Estimates the location of missing landmarks from their bilateral counterparts. |
| pls2B | Performs a two-block partial least squares regression on two sets of Procrustes shape variables. |
| points2plane | Projects a 3D coordinate orthogonally onto a plane. |
| rotaxis3d | Rotates an object around an arbitrary axis in 3D. |
| rotonmat | Rotates a matrix of landmarks by using a rotation determined by two matrices, without scaling to minimise sums of squared distances (scale = FALSE). |
| rotonto | Rotates, translates, and scales one matrix onto another using Procrustes fitting. |
| symmetrize | Creates a perfectly symmetric version of landmarks through reflected relabelling. |
| RRPP (Collyer & Adams, 2018, 2025) | |
| model.comparison | Calculates log-likelihoods to assist in comparative model evaluation or selection (type = “logLik”). |
| stats (R Core Team, 2025) | |
| approx | Returns a list of points which linearly interpolate given data points. |
| cor | Tests for the correlation between samples using the Pearson’s product moment correlation coefficient (method = “pearson”) or the Spearman’s rank correlation coefficient (method = “spearman”). |
| dist | Computes the Euclidean distance between the rows of a data matrix and returns the distance matrix (method = “euclidean”). |
| ecdf | Computes an empirical cumulative distribution function. |
| lm | Carries out a regression, including linear regressions and multiple multivariate regressions. |
| p.adjust | Returns adjusted p-values, controlling the false discovery rate when adjusting for multiplicity (method = “fdr”). |
| prcomp | Performs a principal component analysis. |
| predict | Predicts values based on the results of various model fitting functions. |
| quantile | Produces sample quantiles corresponding to the given probabilities. |
| t.test | Performs a two-sample t-test to compare means. |
| var.test | Performs a two-tailed F-test to compare variances. |

**TABLE S6** Correlation coefficients between the maxillary and mandibular dental arcade shapes for each extant hominine species.

|  | PLS1 | PLS2 |
| --- | --- | --- |
| *H. sapiens* | 0.62316 | 0.56985 |
| *P. troglodytes* | 0.61858 | 0.71281 |
| *P. paniscus* | 0.70144 | 0.52147 |
| *G. gorilla* | 0.65574 | 0.77390 |
| *G. beringei* | 0.49877 | 0.79075 |

**TABLE S7** Means of the natural logarithms of centroid sizes for original and predicted maxillary dental arcades of each extant hominine species, and results of pairwise t-tests comparing the means.

|  | **Original** | **Predicted** | **t-statistic** | ***p*-value** ^a^ |
| --- | --- | --- | --- | --- |
| *H. sapiens* | 5.15212 | 5.13417 | 4.15250 | **<0.001** |
| *P. troglodytes* | 5.32733 | 5.34245 | -5.51817 | **<0.001** |
| *P. paniscus* | 5.14315 | 5.16660 | -6.12350 | **<0.001** |
| *G. gorilla* | 5.52962 | 5.51012 | 5.62275 | **<0.001** |
| *G. beringei* | 5.58643 | 5.59120 | -1.87829 | 0.064 |

a Significant *p*-values are highlighted in bold (*p* < 0.05).

**TABLE S8** Standard deviations of the natural logarithms of centroid sizes for original and predicted maxillary dental arcades of each extant hominine species, and results of pairwise F-tests comparing the standard deviations.

|  | **Original** | **Predicted** | **F-statistic** | ***p*-value** ^a^ |
| --- | --- | --- | --- | --- |
| *H. sapiens* | 0.04903 | 0.04832 | 1.02975 | 0.901 |
| *P. troglodytes* | 0.05200 | 0.05132 | 1.02680 | 0.901 |
| *P. paniscus* | 0.04171 | 0.03905 | 1.14054 | 0.901 |
| *G. gorilla* | 0.08508 | 0.08290 | 1.05333 | 0.901 |
| *G. beringei* | 0.08690 | 0.08414 | 1.06674 | 0.901 |

a Significant *p*-values are highlighted in bold (*p* < 0.05).

**TABLE S9** Means of the Procrustes shape distances between original and predicted maxillary dental arcades for each extant hominine species, compared with the 5% limit of the intraspecific shape distribution.

|  | Procrustes shape distance | 5% Limit |
| --- | --- | --- |
| *H. sapiens* | 0.05172 | 0.05367 |
| *P. troglodytes* | 0.04250 | 0.04877 |
| *P. paniscus* | 0.04826 | 0.05242 |
| *G. gorilla* | 0.05013 | 0.05525 |
| *G. beringei* | 0.05147 | 0.05264 |

**TABLE S10** Means of the Procrustes shape distances between all possible specimen pairs for original and predicted maxillary dental arcades of each extant hominine species, and results of pairwise t-tests comparing the means.

|  | **Original** | **Predicted** | **t-statistic** | ***p*-value** ^a^ |
| --- | --- | --- | --- | --- |
| *H. sapiens* | 0.07732 | 0.04585 | 70.1867 | **<0.001** |
| *P. troglodytes* | 0.07103 | 0.04066 | 126.545 | **<0.001** |
| *P. paniscus* | 0.07496 | 0.03900 | 71.6740 | **<0.001** |
| *G. gorilla* | 0.08345 | 0.05104 | 78.7955 | **<0.001** |
| *G. beringei* | 0.08663 | 0.05126 | 98.6456 | **<0.001** |

a Significant *p*-values are highlighted in bold (*p* < 0.05).

**TABLE S11** Standard deviations of the natural logarithms of centroid sizes for each species, and results of two-tailed F-tests comparing the standard deviations, with the lower triangle showing *p*-values ^a^ and the upper triangle F-statistics. For *A. afarensis*, data is shown for the original maxillary dental arcade sizes and including predictions.

|  | Standard deviation | *A. afarensis* | *A. afarensis*+ | *H. sapiens* | *P. troglodytes* | *P. paniscus* | *G. gorilla* | *G. beringei* |
| --- | --- | --- | --- | --- | --- | --- | --- | --- |
| *A. afarensis* | 0.09781 | — | 1.082 | 3.540 | 3.723 | 5.362 | 1.186 | 1.310 |
| *A. afarensis*+ | 0.10176 | 0.972 | — | 3.832 | 4.030 | 5.804 | 1.284 | 1.418 |
| *H. sapiens* | 0.05198 | **0.004** | **<0.001** | — | 1.052 | 1.515 | 0.335 | 0.370 |
| *P. troglodytes* | 0.05069 | **0.003** | **<0.001** | 0.803 | — | 1.440 | 0.319 | 0.352 |
| *P. paniscus* | 0.04224 | **<0.001** | **<0.001** | 0.177 | 0.231 | — | 0.221 | 0.244 |
| *G. gorilla* | 0.08981 | 0.675 | 0.445 | **<0.001** | **<0.001** | **<0.001** | — | 1.104 |
| *G. beringei* | 0.08546 | 0.618 | 0.288 | **<0.001** | **<0.001** | **<0.001** | 0.675 | — |

a Significant *p*-values are highlighted in bold (*p* < 0.05).

**TABLE S12** Means of the Procrustes shape distances between all possible specimen pairs for each species, and results of two-sample t-tests comparing the means, with the lower triangle showing *p*-values ^a^ and the upper triangle t-statistics. For *A. afarensis*, data is shown only for the original maxillary dental arcade shapes.

|  | Procrustes  shape distance | *A. afarensis* | *H. sapiens* | *P. troglodytes* | *P. paniscus* | *G. gorilla* | *G. beringei* |
| --- | --- | --- | --- | --- | --- | --- | --- |
| *A. afarensis* | 0.08703 | — | 3.277 | 4.987 | 3.842 | 0.374 | 0.264 |
| *H. sapiens* | 0.07745 | **0.003** | — | 14.836 | 3.251 | -22.157 | -21.506 |
| *P. troglodytes* | 0.07245 | **<0.001** | **<0.001** | — | -5.896 | -34.927 | -33.474 |
| *P. paniscus* | 0.07568 | **<0.001** | **0.002** | **<0.001** | — | -17.778 | -17.783 |
| *G. gorilla* | 0.08593 | 0.761 | **<0.001** | **<0.001** | **<0.001** | — | -0.716 |
| *G. beringei* | 0.08625 | 0.793 | **<0.001** | **<0.001** | **<0.001** | 0.547 | — |

a Significant *p*-values are highlighted in bold (*p* < 0.05).

**TABLE S13** Probability of no significant difference (*p* > 0.05), a significant increase (*p* < 0.05, t > 0), and a significant decrease (*p* < 0.05, t < 0) when comparing the means of the Procrustes shape distances between all possible specimen pairs for each extant hominine species with that of *A. afarensis* when including randomly sampled corrected predictions.

|  | **Not significant (%)** | **Significant increase (%)** | **Significant decrease (%)** |
| --- | --- | --- | --- |
| *H. sapiens* | 000.0 | 100.0 | 000.0 |
| *P. troglodytes* | 000.0 | 100.0 | 000.0 |
| *P. paniscus* | 000.0 | 100.0 | 000.0 |
| *G. gorilla* | 038.3 | 057.6 | 004.1 |
| *G. beringei* | 043.3 | 051.4 | 005.3 |

**TABLE S14** Results of the multivariate shape regressions on continent of origin for *H. sapiens* and on sex for each extant hominine species.

|  | **R^2^** | **SS** | **F-value** | ***p*-value** ^a^ |
| --- | --- | --- | --- | --- |
| **Geographical distribution** |  |  |  |  |
| *H. sapiens* | 0.11726 | 0.03593 | 2.03691 | **<0.001** |
| **Sexual dimorphism** |  |  |  |  |
| *H. sapiens* | 0.01053 | 0.00292 | 0.93687 | 0.456 |
| *P. troglodytes* | 0.07962 | 0.02182 | 8.47765 | **<0.001** |
| *P. paniscus* | 0.09647 | 0.01329 | 4.80471 | **<0.001** |
| *G. gorilla* | 0.20137 | 0.07809 | 24.7108 | **<0.001** |
| *G. beringei* | 0.20386 | 0.08160 | 25.3506 | **<0.001** |

a Significant *p*-values are highlighted in bold (*p* < 0.05).

**TABLE S15** Results of the multivariate shape regressions on the natural logarithms of centroid sizes for each species. For *A. afarensis*, data is shown only for the original maxillary dental arcade shapes.

|  | **R^2^** | **SS** | **F-value** | ***p*-value** ^a^ |
| --- | --- | --- | --- | --- |
| *A. afarensis* | 0.06824 | 0.00215 | 0.51268 | 0.951 |
| *H. sapiens* | 0.03498 | 0.01086 | 3.55250 | **0.001** |
| *P. troglodytes* | 0.05712 | 0.01565 | 5.93672 | **<0.001** |
| *P. paniscus* | 0.06743 | 0.00929 | 3.25367 | **0.004** |
| *G. gorilla* | 0.20317 | 0.07879 | 24.9873 | **<0.001** |
| *G. beringei* | 0.20054 | 0.08027 | 24.8331 | **<0.001** |

a Significant *p*-values are highlighted in bold (*p* < 0.05).

**TABLE S16** Probability of a non-significant (*p* > 0.05) or a significant (*p* < 0.05) multivariate shape regression on the natural logarithms of centroid sizes for *A. afarensis* when including randomly sampled corrected predictions, and associated range of R^2^ values.

|  | **Not significant** | **Significant** |
| --- | --- | --- |
| Probability (%) | 97.7 | 02.3 |
| R^2^ (min–max) | 0.01459–0.07614 | 0.06968–0.10089 |

**TABLE S17** Akaike information criterion (AIC) values and their relative likelihood (relLik) for four multivariate shape regression models for each extant hominine species.

|  |  | **ln centroid size** | **sex** | **ln centroid size + sex** | **ln centroid size * sex** |
| --- | --- | --- | --- | --- | --- |
| *H. sapiens* | AIC | -61624 | -61524 | -61705 | -61816 |
|  | relLik | 0 | 0 | 0 | 1 |
| *P. troglodytes* | AIC | -69882 | -69866 | -69986 | -69990 |
|  | relLik | 0 | 0 | 0.18 | 1 |
| *P. paniscus* | AIC | -2949 | -2949 | -2859 | -2767 |
|  | relLik | 0.68 | 1 | 0 | 0 |
| *G. gorilla* | AIC | -68541 | -68588 | -68657 | -68704 |
|  | relLik | 0 | 0 | 0 | 1 |
| *G. beringei* | AIC | -70155 | -70225 | -70355 | -70438 |
|  | relLik | 0 | 0 | 0 | 1 |

**TABLE S18** Means and standard deviations of the natural logarithms of centroid sizes for males and females of each species, the results of two-sample t-tests comparing the means, and the male/female ratios of mean size differences. For *A. afarensis*, data is shown for the original maxillary dental arcade sizes and including predictions.

|  | **Male** | **Female** | **t-statistic** | ***p*-value** ^a^ | **Ratio** |
| --- | --- | --- | --- | --- | --- |
| *A. afarensis* | 5.311 ± 0.046 | 5.145 ± 0.049 | 5.16989 | **0.003** | 1.03216 |
| *A. afarensis*+ | 5.334 ± 0.062 | 5.173 ± 0.062 | 6.58006 | **<0.001** | 1.03105 |
| *H. sapiens* | 5.156 ± 0.051 | 5.129 ± 0.049 | 2.46401 | **0.019** | 1.00520 |
| *P. troglodytes* | 5.348 ± 0.050 | 5.307 ± 0.042 | 4.46327 | **<0.001** | 1.00781 |
| *P. paniscus* | 5.159 ± 0.048 | 5.134 ± 0.033 | 2.06168 | **0.047** | 1.00498 |
| *G. gorilla* | 5.598 ± 0.052 | 5.444 ± 0.039 | 16.6795 | **<0.001** | 1.02816 |
| *G. beringei* | 5.652 ± 0.039 | 5.498 ± 0.036 | 20.6592 | **<0.001** | 1.02799 |

a Significant *p*-values are highlighted in bold (*p* < 0.05).

**TABLE S19** Means of the geometrically manipulated Procrustes form and shape distances between all possible pairs of males and females for each species, and results of two-sample t-tests comparing the means, with the lower triangle showing *p*-values ^a^ and the upper triangle t-statistics. For *A. afarensis*, data is shown only for the original maxillary dental arcade shapes.

|  | Procrustes distance | *A. afarensis* | *H. sapiens* | *P. troglodytes* | *P. paniscus* | *G. gorilla* | *G. beringei* |
| --- | --- | --- | --- | --- | --- | --- | --- |
| Form |  |  |  |  |  |  |  |
| *A. afarensis* | 0.24343 | — | 11.876 | 5.395 | 4.341 | -7.279 | -7.656 |
| *H. sapiens* | 0.06703 | **<0.001** | — | -37.869 | -20.189 | -82.465 | -97.274 |
| *P. troglodytes* | 0.16341 | **<0.001** | **<0.001** | — | -2.330 | -56.180 | -67.499 |
| *P. paniscus* | 0.17582 | **<0.001** | **<0.001** | **0.022** | — | -30.377 | -32.734 |
| *G. gorilla* | 0.35274 | **<0.001** | **<0.001** | **<0.001** | **<0.001** | — | -1.309 |
| *G. beringei* | 0.35760 | **<0.001** | **<0.001** | **<0.001** | **<0.001** | 0.191 | — |
| Shape |  |  |  |  |  |  |  |
| *A. afarensis* | 0.03487 | — | 5.804 | 1.344 | 0.258 | -5.347 | -5.621 |
| *H. sapiens* | 0.01181 | **<0.001** | — | -34.396 | -20.855 | -67.252 | -79.181 |
| *P. troglodytes* | 0.02954 | 0.209 | **<0.001** | — | -4.196 | -44.374 | -54.914 |
| *P. paniscus* | 0.03382 | 0.799 | **<0.001** | **<0.001** | — | -20.337 | -22.267 |
| *G. gorilla* | 0.05617 | **<0.001** | **<0.001** | **<0.001** | **<0.001** | — | -1.563 |
| *G. beringei* | 0.05719 | **<0.001** | **<0.001** | **<0.001** | **<0.001** | 0.136 | — |

a Significant *p*-values are highlighted in bold (*p* < 0.05).

**TABLE S20** Probability of no significant difference (*p* > 0.05), a significant increase (*p* < 0.05, t > 0), and a significant decrease (*p* < 0.05, t < 0) when comparing the means of the geometrically manipulated Procrustes form and shape distances between all possible pairs of males and females for each extant hominine species with that of *A. afarensis* when including randomly sampled corrected predictions.

|  | **Not significant (%)** | **Significant increase (%)** | **Significant decrease (%)** |
| --- | --- | --- | --- |
| **Form** | |  |  |
| *H. sapiens* | 000.0 | 100.0 | 000.0 |
| *P. troglodytes* | 000.0 | 100.0 | 000.0 |
| *P. paniscus* | 037.1 | 062.9 | 000.0 |
| *G. gorilla* | 000.0 | 000.0 | 100.0 |
| *G. beringei* | 000.0 | 000.0 | 100.0 |
| **Shape** | |  |  |
| *H. sapiens* | 000.2 | 099.8 | 000.0 |
| *P. troglodytes* | 018.4 | 000.1 | 081.5 |
| *P. paniscus* | 005.2 | 000.0 | 094.8 |
| *G. gorilla* | 000.0 | 000.0 | 100.0 |
| *G. beringei* | 000.0 | 000.0 | 100.0 |

**TABLE S21** Means of the two-dimensional Procrustes shape distances between all possible specimen pairs for each species, and results of two-sample t-tests comparing the means, with the lower triangle showing *p*-values ^a^ and the upper triangle t-statistics. For *A. afarensis*, data is shown only for the original maxillary dental arcade shapes.

|  | Procrustes  shape distance | *A. afarensis* | *H. sapiens* | *P. troglodytes* | *P. paniscus* | *G. gorilla* | *G. beringei* |
| --- | --- | --- | --- | --- | --- | --- | --- |
| *A. afarensis* | 0.07123 | — | 2.230 | 2.581 | 2.463 | -1.261 | -1.018 |
| *H. sapiens* | 0.06448 | **0.048** | — | 3.000 | 1.369 | -26.224 | -23.296 |
| *P. troglodytes* | 0.06341 | **0.027** | **0.006** | — | -0.485 | -29.204 | -26.091 |
| *P. paniscus* | 0.06369 | **0.031** | 0.214 | 0.628 | — | -18.866 | -17.275 |
| *G. gorilla* | 0.07505 | 0.249 | **<0.001** | **<0.001** | **<0.001** | — | 1.590 |
| *G. beringei* | 0.07432 | 0.338 | **<0.001** | **<0.001** | **<0.001** | 0.152 | — |

a Significant *p*-values are highlighted in bold (*p* < 0.05).

**TABLE S22** Probability of no significant difference (*p* > 0.05), a significant increase (*p* < 0.05, t > 0), and a significant decrease (*p* < 0.05, t < 0) when comparing the means of the two-dimensional Procrustes shape distances between all possible specimen pairs for each extant hominine species with that of *A. afarensis* when including randomly sampled corrected predictions.

|  | **Not significant (%)** | **Significant increase (%)** | **Significant decrease (%)** |
| --- | --- | --- | --- |
| *H. sapiens* | 000.0 | 100.0 | 000.0 |
| *P. troglodytes* | 000.0 | 100.0 | 000.0 |
| *P. paniscus* | 000.0 | 100.0 | 000.0 |
| *G. gorilla* | 058.3 | 021.4 | 020.3 |
| *G. beringei* | 058.3 | 028.8 | 012.9 |

**TABLE S23** Mandibular corpus heights for *A. afarensis* specimens from various localities.

| **Specimen** | **Geological**  **age (Ma)** ^a^ | **Mandibular corpus height (mm)** ^b^ | | **Reference for measurement** |
| --- | --- | --- | --- | --- |
|  |  | **M_1_** | **M_2_** |  |
| **Hadar (Ethiopia)** | | | | |
| A.L. 145-35 | 3.42–3.38 | 27.8 | — | White & Johanson (1982) |
| A.L. 188-1 | 3.216–3.20 | — | 34.3 | White & Johanson (1982) |
| A.L. 198-1 | 3.42–3.38 | 31.1 | 30.8 | White & Johanson (1982) |
| A.L. 198-22 | 3.42–3.38 | — | 34.0 | Kimbel et al. (2004) |
| A.L. 207-13 | 3.236–3.216 | 28.4 | 25.3 | White & Johanson (1982) |
| A.L. 225-8 | 3.42–3.38 | 31.1 | 28.1 | Kimbel et al. (2004) |
| A.L. 228-2 | 3.35–3.30 | 31.8 | — | Kimbel et al. (2004) |
| A.L. 266-1 | 3.35–3.30 | 31.5 | 27.6 | White & Johanson (1982) |
| A.L. 277-1 | 3.38–3.35 | 37.0 | — | White & Johanson (1982) |
| A.L. 288-1i | Closer to 3.20 | 30.0 | 27.6 | White & Johanson (1982) |
| A.L. 315-22 | 3.216–3.20 | 29.7 | 28.0 | Kimbel et al. (2004) |
| A.L. 330-5 | 3.30–3.256 | 31.1 | 28.3 | Kimbel et al. (2004) |
| A.L. 333w-1ab | 3.236–3.216 | 35.3 | 32.4 | White & Johanson (1982) |
| A.L. 333w-12 | 3.236–3.216 | 30.6 | — | White & Johanson (1982) |
| A.L. 333w-60 | 3.236–3.216 | 38.4 | 35.4 | White & Johanson (1982) |
| A.L. 400-1a | 3.38–3.35 | 35.4 | — | White & Johanson (1982) |
| A.L. 417-1a | 3.35–3.30 | 36.0 | 32.8 | Kimbel et al. (2004) |
| A.L. 418-1 | 3.35–3.30 | — | 36.0 | Kimbel et al. (2004) |
| A.L. 433-1ab | 3.216–3.20 | 35.0 | — | Kimbel et al. (2004) |
| A.L. 436-1 | 3.38–3.35 | — | 26.0 | Kimbel et al. (2004) |
| A.L. 437-1 | 3.12–2.96 | 40.0 | — | Kimbel et al. (2004) |
| A.L. 437-2 | 3.12–2.96 | 38.5 | 37.0 | Kimbel et al. (2004) |
| A.L. 438-1g | 3.12–2.96 | 41.3 | 37.1 | Kimbel et al. (2004) |
| A.L. 444-2b | 3.12–2.96 | 41.2 | 37.6 | Kimbel et al. (2004) |
| A.L. 620-1 | 3.236–3.216 | 36.2 | 34.5 | Kimbel et al. (2004) |
| A.L. 822-1 | Closer to 3.12 | 36.1 | 31.7 | Measured from CT scan |
| A.L. 1045-1 | 3.38–3.35 | 33.0 | 31.2 | Measured from CT scan |
| A.L. 1180-1 | 3.30–3.256 | 29.8 | 29.5 | Measured from CT scan |
| A.L. 1496-1 | 3.42–3.38 | 36.1 | 34.0 | Measured from CT scan |
| **Laetoli (Tanzania)** | | | | |
| L.H. 4 | 3.85–3.63 | 31.4 | 29.5 | White (1977) |
| L.H. 29 | 3.85–3.63 | — | 38.4 | Harrison (2011) |
| **Maka (Ethiopia)** | | | | |
| MAK-VP-1/2 | ca. 3.42 | — | 32.6 | White et al. (2000) |
| MAK-VP-1/12 | ca. 3.42 | 30.5 | 29.6 | White et al. (2000) |
| **Dikika (Ethiopia)** | | | | |
| DIK-2-1 | 3.45–3.42 | 38.5 | — | Alemseged et al. (2005) ^c^ |
| **Woranso-Mille (Ethiopia)** | | | | |
| NFR-VP-1/29 | 3.33–3.207 | 37.9 | 34.0 | Haile-Selassie et al. (2016) |
| LDD-VP-1/167 | 3.42–3.33 | 29.4 | — | Melillo et al. (2021) |

a Obtained from Lourens et al. (2004), Campisano (2007), and Deino (2011). Geological age ranges in White et al. (2000), Alemseged et al. (2005), and Melillo et al. (2021) are adapted to match the 3.42 Ma age of the Sidi Hakoma Tuff in Campisano (2007).
b When both sides were measured, the value shown is the average of the left and right side.
c Estimated from Figure 5 in Alemseged et al. (2005).

**REFERENCES**

Adams, D. C., Collyer, M. L., Kaliontzopoulou, A., & Baken, E. K. (2025). *geomorph: Geometric morphometric analyses of 2D/3D landmark data*. [https://CRAN.R-project.
org/package=geomorph](https://CRAN.R-project.org/package=geomorph)

Alemseged, Z., Wynn, J. G., Kimbel, W. H., Reed, D., Geraads, D., & Bobe, R. (2005). A new hominin from the Basal Member of the Hadar Formation, Dikika, Ethiopia, and its geological context. *Journal of Human Evolution*, *49*(4), 499–514. [https://doi.org/10.
1016/j.jhevol.2005.06.001](https://doi.org/10.1016/j.jhevol.2005.06.001)

Baken, E. K., Collyer, M. L., Kaliontzopoulou, A., & Adams, D. C. (2021). geomorph v4.0 and gmShiny: Enhanced analytics and a new graphical interface for a comprehensive morphometric experience. *Methods in Ecology and Evolution*, *12*(12), 2355–2363. <https://doi.org/10.1111/2041-210X.13723>

Campisano, C. J. (2007). *Tephrostratigraphy and hominin paleoenvironments of the Hadar Formation, Afar Depression, Ethiopia* [Doctoral dissertation, Rutgers University]. RU Community Repository. <https://doi.org/doi:10.7282/T3NS0V99>

Collyer, M. L., & Adams, D. C. (2018). RRPP: An R package for fitting linear models to high‐dimensional data using residual randomization. *Methods in Ecology and Evolution*, *9*(7), 1772–1779. <https://doi.org/10.1111/2041-210X.13029>

Collyer, M. L., & Adams, D. C. (2025). *RRPP: Linear model evaluation with randomized residuals in a permutation procedure*. <https://CRAN.R-project.org/package=RRPP>

Del Re, A.C., & Hoyt, W.T. (2024). *MAd: Meta-analysis with mean differences*. <https://CRAN.R-project.org/package=MAd>

Deino, A. L. (2011). ^40^Ar/^39^Ar dating of Laetoli, Tanzania. In T. Harrison (Ed.), *Paleontology and geology of Laetoli: Human evolution in context* (Vol. 1, pp. 77–97). Springer. <https://doi.org/10.1007/978-90-481-9956-3_4>

Drapeau, M. S. M., Ward, C. V., Kimbel, W. H., Johanson, D. C., & Rak, Y. (2005). Associated cranial and forelimb remains attributed to *Australopithecus afarensis* from Hadar, Ethiopia. *Journal of Human Evolution*, *48*(6), 593–642. [https://doi.org/10.1016/j.jhevol.
2005.02.005](https://doi.org/10.1016/j.jhevol.2005.02.005)

Haile-Selassie, Y., Melillo, S. M., Ryan, T. M., Levin, N. E., Saylor, B. Z., Deino, A. L., Mundil, R., Scott, G., Alene, M., & Gibert, L. (2016). Dentognathic remains of *Australopithecus afarensis* from Nefuraytu (Woranso-Mille, Ethiopia): Comparative description, geology, and paleoecological context. *Journal of Human Evolution*, *100*, 35–53. [https://doi.org/
10.1016/j.jhevol.2016.08.003](https://doi.org/10.1016/j.jhevol.2016.08.003)

Hanegraef, H., & Spoor, F. (2025). Morphological variation of the *Australopithecus afarensis* maxilla. *Journal of Human Evolution*, *201*, Article 103651. [https://doi.org/10.
1016/j.jhevol.2025.103651](https://doi.org/10.1016/j.jhevol.2025.103651)

Harrison, T. (2011). Hominins from the Upper Laetolil and Upper Ndolanya Beds, Laetoli. In T. Harrison (Ed.), *Paleontology and geology of Laetoli: Human evolution in context* (Vol. 2, pp. 141–188). Springer. <https://doi.org/10.1007/978-90-481-9962-4_7>

Johanson, D. C., Lovejoy, C. O., Kimbel, W. H., White, T. D., Ward, S. C., Bush, M. E., Latimer, B. M., & Coppens, Y. (1982). Morphology of the Pliocene partial hominid skeleton (A.L. 288‐1) from the Hadar formation, Ethiopia. *American Journal of Physical Anthropology*, *57*(4), 403–451. <https://doi.org/10.1002/ajpa.1330570403>

Kimbel, W. H., Rak, Y., & Johanson, D. C. (2004). *The skull of* Australopithecus afarensis. Oxford University Press. <https://doi.org/10.1093/oso/9780195157062.001.0001>

Kimbel, W. H., & Delezene, L. K. (2009). “Lucy” redux: A review of research on *Australopithecus afarensis*. *American Journal of Physical Anthropology*, *140*(S49), 2–48. <https://doi.org/10.1002/ajpa.21183>

Lourens, L., Hilgen, F., Shackleton, N. J., Laskar, J., & Wilson, D. (2004). The Neogene period. In F. Gradstein, J. G. Ogg, & G. Smith (Eds.), *A geologic time scale 2004* (pp. 409–440). Cambridge University Press. <https://doi.org/10.1017/CBO9780511536045.022>

Melillo, S. M., Gibert, L., Saylor, B. Z., Deino, A. L., Alene, M., Ryan, T. M., & Haile-Selassie, Y. (2021). New Pliocene hominin remains from the Leado Dido’a area of Woranso-Mille, Ethiopia. *Journal of Human Evolution*, *153*, Article 102956. [https://doi.org/10.
1016/j.jhevol.2021.102956](https://doi.org/10.1016/j.jhevol.2021.102956)

R Core Team (2025). *R: A language and environment for statistical computing*. R Foundation for Statistical Computing.

Robinson, C. A. (2003). *Extant hominoid and australopith mandibular morphology; assessing alpha taxonomy and phylogeny in hominoids using mandibular characters* (Publication No. 3089322) [Doctoral dissertation, New York University]. ProQuest Dissertations & Theses Global.

Ripley, B. D., & Venables, W. N. (2025). *MASS: Support functions and datasets for Venables and Ripley’s MASS*. [https://CRAN.R-project.org/package=MASS](https://cran.r-project.org/package=MASS).

Schlager, S. (2017). Morpho and Rvcg – Shape analysis in R: R-packages for geometric morphometrics, shape analysis and surface manipulations. In G. Zheng, S. Li, & G. Szekely (Eds.), *Statistical shape and deformation analysis: Methods, implementation and applications* (pp. 217–256). Academic Press. <https://doi.org/10.1016/B978-0-12-810493-4.00011-0>

Schlager, S. (2025). *Morpho: Calculations and visualisations related to geometric morphometrics*. <https://CRAN.R-project.org/package=Morpho>

Venables, W. N., & Ripley, B. D. (2002). *Modern applied statistics with S*. Springer. [https://doi.
org/10.1007/978-0-387-21706-2](https://doi.org/10.1007/978-0-387-21706-2)

White, T. D. (1977). New fossil hominids from Laetolil, Tanzania. *American Journal of Physical Anthropology*, *46*(2), 197–230. <https://doi.org/10.1002/ajpa.1330460203>

White, T. D., & Johanson, D. C. (1982). Pliocene hominid mandibles from the Hadar Formation, Ethiopia: 1974–1977 collections. *American Journal of Physical Anthropology*, *57*(4), 501–544. <https://doi.org/10.1002/ajpa.1330570405>

White, T. D., Suwa, G., Simpson, S. W., & Asfaw, B. (2000). Jaws and teeth of *Australopithecus afarensis* from Maka, Middle Awash, Ethiopia. *American Journal of Physical Anthropology*, *111*(1), 45–68. [https://doi.org/10.1002/(SICI)1096-8644(200001)111:1<45::AID-AJPA4>3.0.CO;2-I](https://doi.org/10.1002/(SICI)1096-8644(200001)111:1%3c45::AID-AJPA4%3e3.0.CO;2-I)
